# An *Adamts2* Knock-In Model of Dermatosparaxis Ehlers–Danlos Syndrome Reveals Defective Collagen Maturation

**DOI:** 10.3390/biomedicines14091928

**Published:** 2026-08-27

**Authors:** Taylor Petrucci-Nelson, Amy Weintraub, Matthew Huff, Emma Mach, Cortney Gensemer, Cara Virgin, Madalyn Osterhaus, Kathryn Byerly, Erika Bistran, Sydney Severance, Brian Loizzi, Jan Guz, Fu Lei Tang, Molly Griggs, Sunil Patel, Russell A. Norris

**Affiliations:** 1 Department of Regenerative Medicine and Cell Biology, Medical University of South Carolina, Charleston, SC 29425, USA; nelson.taylorann@gmail.com (T.P.-N.); weintrau@musc.edu (A.W.); huffmat@musc.edu (M.H.); emach1022@outlook.com (E.M.); cortney.gensemer@chroniclebio.com (C.G.); carapvirgin@gmail.com (C.V.); osterhausmadalyn@gmail.com (M.O.); byerlyk@musc.edu (K.B.); bistran@musc.edu (E.B.); sydneyseverance@icloud.com (S.S.); loizzi@musc.edu (B.L.); griggsma@musc.edu (M.G.); 2 Department of Neurosurgery, Medical University of South Carolina, Charleston, SC 29425, USA; patels@musc.edu; 3 Department of Comparative Medicine Transgenic and Gene (TGE) Core, Medical University of South Carolina, Charleston, SC 29425, USA; guzj@musc.edu (J.G.); fulei@musc.edu (F.L.T.)

**Keywords:** dermatosparaxis Ehlers–Danlos syndrome, ADAMTS2, extracellular matrix, collagen fibrillogenesis, single-nucleus RNA sequencing, skin fragility

## Abstract

**Background/Objectives:** Dermatosparaxis Ehlers–Danlos syndrome (dEDS) is a rare autosomal recessive connective tissue disorder caused by biallelic pathogenic variants in *ADAMTS2*, which encodes the primary N-proteinase responsible for fibrillar procollagen processing. Although defective procollagen cleavage is the defining molecular feature of dEDS, how ADAMTS2 deficiency disrupts extracellular matrix (ECM) organization and tissue integrity remains incompletely understood. Here we characterized the structural, molecular, and cellular consequences of a knock-in *Adamts2* mouse model harboring a disease-associated variant and assessed its phenotypic and mechanistic resemblance to human dEDS. **Methods:** We generated Adamts2^Q226*^ mice carrying a variant analogous to a human dEDS-causing mutation. Skin from homozygous, heterozygous, and control animals was evaluated using histologic, ultrastructural, biochemical, digital pathology, and single-nucleus RNA-sequencing approaches. Pathway enrichment analyses and the computational tool CellChat were used to infer altered molecular programs and changes in intercellular communication. **Results:** Homozygous knock-in mice exhibited near-complete loss of dermal ADAMTS2 protein expression, impaired type I procollagen processing, disrupted dermal architecture, and irregular hieroglyphic collagen fibrils characteristic of dEDS. Digital pathology demonstrated reduced collagen bulk, diminished assembled and total collagen, increased fine collagen, and loss of mature collagen architecture, with intermediate changes in heterozygous animals. Single-nucleus RNA sequencing identified fibroblasts as the most affected population, with coordinated downregulation of collagen, microfibrillar, and other ECM-associated genes. Pathway analyses implicated altered ECM organization, receptor-linked signaling, cytoskeletal regulation, protein processing, and metabolism, while CellChat inferred widespread reductions in intercellular communication. **Conclusions:** ADAMTS2 deficiency causes fibroblast-enriched transcriptional remodeling, impaired collagen processing and ECM maturation, and disrupted tissue-wide cellular communication. This model provides a translational platform for studying dEDS pathogenesis and strategies to restore ECM homeostasis.

## 1. Introduction

Ehlers–Danlos Syndromes (EDS) are a group of heritable connective tissue disorders characterized by skin hyperextensibility, joint hypermobility, and tissue fragility [1]. Dermatosparaxis Ehlers–Danlos syndrome (dEDS) is one of the 12 rare EDS subtypes and is clinically characterized by profound skin fragility, sagging or redundant skin, easy bruising, atrophic scarring, and umbilical hernias, together with a range of multisystem manifestations extending beyond the skin [1]. This subtype is one of the rarest EDS subtypes affecting <1,000,000 individuals worldwide and is caused by biallelic pathogenic variants in *ADAMTS2*, which encodes procollagen I N-proteinase [1,2]. This enzyme is essential for removal of N-terminal propeptides from fibrillar procollagens, a prerequisite for normal fibrillogenesis [3]. Loss of ADAMTS2 function leads to defective collagen processing and distinctive “hieroglyphic” appearance of collagen fibrils upon ultrastructural analysis [4]. Despite identification of the genetic cause, the mechanisms linking ADAMTS2 deficiency to extracellular matrix (ECM) disorganization remain unknown.

*Adamts2* knockout mice reproduce key aspects of dEDS, including skin fragility and abnormal collagen fibrils [5], but a knock-in model reflecting a human pathogenic variant has not been reported. Such a model would provide a unique opportunity to define how ADAMTS2 regulates collagen maturation in vivo, from procollagen processing and fibril assembly to higher-order ECM organization and downstream cellular responses, while establishing a translational platform for studying dEDS pathogenesis [6]. In this study, we generated and characterized a knock-in allele for *Adamts2*, targeting the amino acid position corresponding to the human variant as a potentially genetically accurate mouse model of dEDS. Utilizing histology, immunohistochemistry, transmission electron microscopy (TEM), Western blot, and digital pathology, we demonstrate impaired collagen processing, disrupted ECM organization, and defective collagen assembly in homozygous mice. Single-nuclei RNA sequencing and CellChat analyses further inferred fibroblast-specific transcriptional changes, including downregulation of many structural ECM genes such as *Collagen VI* and *Fibrillin-1*, which were validated at the protein level. Together, these data establish the first knock-in model of dEDS and provide new insight into how loss of ADAMTS2 drives collagen and ECM pathology.

## 2. Materials and Methods

### 2.1. Ethics Statement

The animal study protocol was approved by the Institutional Animal Care and Use Committee (IACUC) of the Medical University of South Carolina (IACUC-2020-00956, date of most recent approval 24 March 2026).

### 2.2. Adamts2 Expression

Developmental *Adamts2* expression was evaluated using publicly available RNA in situ hybridization data from the GenePaint developmental gene-expression resource (https://gp3.mpg.de/results/adamts2, accessed on 1 June 2025). A representative sagittal section from an embryonic day 14.5 (E14.5) mouse embryo was used for analyses. The GenePaint probe sequence corresponding to the *Adamts2* in situ hybridization experiment is provided through the website. Embryonic tissues in the GenePaint dataset were sectioned at 25 µm, hybridized at 64 °C, washed at 62 °C, and imaged at a maximum resolution of 1.64 µm/pixel, according to the source protocol. Adult human ADAMTS2 expression across tissues, sexes, cell populations, exons, and exon–exon junctions was evaluated using publicly available Genotype-Tissue Expression (GTEx; https://gtexportal.org/home/ accessed on 4 September 2025) bulk and single-cell/single-nucleus transcriptomic datasets.

### 2.3. Generation of Adamts2^Q226*^ Mouse Model

To model the ADAMTS2 c.673C > T (p.Gln225Ter) variant linked to dEDS, as reported by Colige et. al. [7] *Adamts2*^Q226*^ mice were generated via CRISPR-mediated knock-in of the Adamts2^Q226*^ mutation at the mouse *Adamts2* locus in the Medical University of South Carolina (MUSC) Transgenic & Genome Editing (TGE) Core. A single guide RNA (gRNA) (5′-CCCTCCTGTGAGCGAACCAC) targeting codon Q226 in exon 3 of mouse *Adamts2* was used. A single-stranded donor DNA (ssODN) (5′-CATGTGGTATATCGCCGACCGCCCACTCCCAAGCCCCCTCCTGTGAGCGAgCCctAGGCTCTGGACACAGGTAAGGCTTCTGCCGAGGGTAGGCTAGGTGGGGAAC), with the mutation (bold) converting the glutamine codon (CAG) to a stop codon (TAG) by replacing “C” with “T,” was introduced. Two additional silent nucleotide changes (underline) were introduced upstream to prevent CRISPR re-cutting of the modified allele. F0 founders were screened using mutant PCR (WT-F: 5′-CTTGCTGCTCTTCCTCCTCT-3′, Mut-R: 5′-TGTGTCCAGAGCCTAGGGC-3′), and the Adamts2 exon 3 region was sequenced using WT-F: 5′-CTTGCTGCTCTTCCTCCTCT-3′ and WT-R: 5′-CCGACTCCTTCCTAAGCCTC-3′ via Sanger sequencing. Correctly targeted founders were bred with C57BL/6J mice, and germline transmission of the Adamts2^Q226*^ mutation was confirmed by PCR genotyping and Sanger sequencing. For accurate nomenclature purposes, the full strain is listed as C57BL/6J-Adamts2^Q226*-em1Norris^/MUSC. The full strain designation, C57BL/6J-Adamts2^Q226*-em1Norris^/MUSC, denotes a C57BL/6J-background mouse carrying a CRISPR-engineered Adamts2^Q226*^ nonsense variant in *Adamts2*; ‘em1Norris/MUSC’ identifies the first endonuclease-mediated allele generated by the Norris laboratory at the Medical University of South Carolina. For clarity, this allele is referred to hereafter as *Adamts2*^Q226*^. A total of 3 founder lines were generated with correct targeting validated by PCR genotyping and Sanger sequencing.

### 2.4. Tissue Collection and Processing

Dorsal skin was dissected from 1 to 2-month-old mice and divided for downstream processing. Histology samples were fixed overnight in 10% zinc formalin followed by ethanol dehydration and paraffin embedding per standard protocols [8]. Tissues for TEM were fixed in 2.5% glutaraldehyde per standard protocols. For snRNA-seq, FFPE blocks were sectioned at 25 µm, and scrolls were collected using a sterile scalpel. Skin for Western analyses was lysed in radioimmunoprecipitation assay buffer, minced, sonicated, centrifuged, and stored at −80 °C per standard protocols [8].

### 2.5. Immunohistochemistry

After euthanasia, skin was excised and fixed as described above. Paraffin-embedded tissues were sectioned and mounted on charged slides, deparaffinized in xylene, and rehydrated through graded alcohols. Antigen retrieval was performed by pressure heating for 1 min at high pressure in a 0.93% citric acid solution (Vector Laboratories, Cat. No. H-3300; Newark, NJ, USA). Sections were blocked with 1% bovine serum albumin (Invitrogen, Cat. No. B4387, Carlsbad, CA, USA) for 1 h at room temperature and incubated with primary antibodies for 1 h at room temperature. Primary antibodies included Collagen I Telo (1:250; previously described [8], Fibrillin-1 (Abbiotec; Cat. No. 251526, 1:250; San Diego, CA, USA), and Collagen VI (Rockland Immunochemicals; Cat. No. 600-401-108-0.1, 1:100; Limerick, PA, USA). Appropriate fluorophore-conjugated secondary antibodies (Invitrogen; Alexa Fluor 488 or Cy5) were used at 1:100. Nuclei were counterstained with Hoechst (Life Technologies; Cat. No. H3569, 1:10,000; Carlsbad, CA, USA) and slides were mounted using SlowFade Gold Antifade Reagent (Invitrogen; Cat. No. S36936; Carlsbad, CA, USA). Images were acquired using an Echo Revolve microscope (Discover Echo Inc., San Diego, CA, USA). Immunohistochemical analyses were performed on independent biological samples from *n* = 4 mice per genotype. These experiments were used primarily for spatial and qualitative validation of protein-expression patterns.

### 2.6. Western Analyses

Following euthanasia and tissue collection, dorsal skin was homogenized in radioimmunoprecipitation assay (RIPA) buffer, minced with spring scissors, sonicated, and centrifuged (10 min at 17,000× *g* at 4 °C). Protein lysates were analyzed by Western blot with investigators blinded to genotype according to previously described protocols [9]. Membranes were incubated with Collagen I Telo (1:1000; previously described [8] followed by goat α-Rabbit IgG-HRP secondary antibody (Sigma Cat. No. A8275; 1:7500; Saint Louis, MO, USA). Signal was detected using SuperSignal West Fempto Maximum Sensitivity Substrate (Thermo Fisher Scientific/Pierce Cat. No. 34096; Waltham, MA, USA). Western blot analyses were performed using independent skin lysates from *n* = 3 mice per genotype. Densitometric measurements were normalized to total protein visualized by Ponceau staining, and the ratio of processed to unprocessed Collagen I was calculated for each biological replicate. Differences among the three genotypes were evaluated using one-way ANOVA followed by Tukey’s multiple-comparisons test, with *p* < 0.05 considered statistically significant.

### 2.7. Transmission Electron Microscopy (TEM)

Skin samples were harvested and immediately fixed overnight at 4 °C in 2.5% glutaraldehyde (Fisher Scientific # O2957-1; Waltham, MA, USA). Following fixation, tissues were transferred to 1× phosphate-buffered saline and processed for transmission electron microscopy by the University of South Carolina Electron Microscopy Core Facility per standard protocols [9]. TEM analyses were performed as a descriptive ultrastructural assessment using independent wild-type (*n* = 3) and homozygous Adamts2^Q226*/Q226*^ (*n* = 6) skin samples. Because the purpose was to determine whether the characteristic “hieroglyphic” collagen-fibril morphology of dEDS was reproduced in the homozygous knock-in model, these data were evaluated qualitatively and were not subjected to inferential statistical testing. Heterozygous samples were not available for TEM analysis.

### 2.8. Quantitative Fibrosis Profiling

Quantitative digital pathology was performed on independent Masson’s trichrome-stained dorsal skin samples from *n* = 3 mice per genotype using the FibroNest platform (PharmaNest, Inc., Princeton, NJ, USA). FibroNest is a computational image-analysis platform that performs high-content quantification of collagen morphology and tissue architecture. The algorithm generates 336 quantitative fibrosis traits (qFTs) defined by image-derived quantitative fibrosis features/parameters. These features characterize collagen abundance, fiber morphology, maturation, spatial organization, and tissue architecture and include measurements of collagen area and density, fiber number, length and thickness, branching complexity, orientation, heterogeneity, directionality, and related higher-order structural parameters. FibroNest further classifies collagen into fine collagen, representing thinner and less assembled collagen structures, and assembled collagen, representing larger, more organized collagen fiber structures identified by the platform’s segmentation and morphometric algorithms. Total collagen represents the combined collagen signal quantified within the analyzed tissue region. The complete list of qFTs, their definitions, and individual sample values is provided in Table S1 and has been described previously [10,11]. Statistical analyses were performed using GraphPad Prism (version 10, GraphPad Software, San Diego, CA, USA). Comparisons between three genotypes per composite score were made using parametric, ordinary one-way analysis of variance (ANOVA) followed by relevant post hoc tests. Statistical significance was defined as *p* < 0.05. All individual qFTs and corresponding composite scores used in this analysis are reported in Table S1.

### 2.9. Single-Nucleus RNA Sequencing (snRNA-Seq) from FFPE Skin

Single-nucleus RNA sequencing included *n* = 4 biological samples per genotype. This sample size was selected to provide independent animal-level biological replication while enabling high-dimensional, cell-type-resolved analysis across thousands of nuclei per condition. Because the study was designed primarily as a discovery-oriented characterization of a newly generated rare-disease mouse model, and because tissue availability from homozygous animals was limited by the severity of the phenotype, a formal prospective power calculation was not performed. Instead, biological replication was prioritized at the animal level, with large numbers of nuclei contributing to cell-type-specific transcriptional profiling. Two publicly available wild-type skin samples from the Tabula Muris resource (https://figshare.com/projects/TabulaMurisTranscriptomiccharacterizationof20organs_and_tissues_from_Mus_musculus_at_single_cell_resolution/27733, accessed on 4 March 2025) were incorporated to balance biological sample numbers across groups; Harmony was used to mitigate batch effects prior to clustering and downstream analysis [12].

Nuclei were isolated from FFPE skin sections of *Adamts2^+/+^* and homozygous Adamts2^Q226*/Q226*^ mice using the 10× Genomics (Pleasanton, CA, USA) protocol for FFPE Tissue Sections for Chromium Fixed RNA Profiling (CG000632), with minor adaptations for mouse skin. Following isolation, nuclei were pelleted (500× *g*, 10 min, 4 °C), washed in 1× nuclei buffer containing RNase inhibitor, and resuspended in 1× nuclei buffer. An aliquot was stained with trypan blue and counted on an automated counter to determine the concentration of intact nuclei, and the input was adjusted according to the Chromium Fixed RNA Profiling protocol recommendations. Single-nucleus gene expression libraries were prepared using the Chromium Next GEM Single-Cell Fixed RNA Profiling (Gene Expression Flex) chemistry for mouse, including the Chromium Next GEM Single-Cell Fixed RNA Sample Preparation Kit (PN-1000414; 10X Genomics), following the manufacturer’s user guide (CG000691).

### 2.10. snRNA-Seq Data Processing and Analysis

Raw FASTQ files from mouse samples were aligned to the mouse genome (version Mm10) and counted using 10× Genomics Cell Ranger (version 8.0.1)’s “count” command. Cell bender (version 1.0.3) predicted and removed ambient RNA contamination, using default settings [13]. These filtered results were read as input in a Seurat (version 5.1.0) pipeline [14]. The percentage of mitochondrial transcripts was calculated for each nucleus, and nuclei exceeding the predefined mitochondrial-content threshold were excluded during quality control. Cells containing 10% or more mitochondrial genes, fewer than 150 detected genes, or more than 10,000 unique molecular identifier (UMI) counts were filtered out.

Following filtering and the addition of the Tabula Muris samples, the Seurat object was integrated, identifying 6420 gene features prior to data integration. A principal component analysis (PCA) was run on the data, batch effects were corrected using Harmony (version 1.2.1), and the cells were clustered with a resolution of 0.6 to ensure unique clusters were properly identified [15]. The clustered data were converted to a single-cell experiment object using SingleCellExperiment (version 1.26.0) to identify singlets and doublets through ScDblFinder (version 1.18.0), using the data from the RNA assay [16,17]. All cells identified as doublets were filtered out of the data, and the same clustering workflow described above was rerun on the filtered dataset. Each cluster was then assigned a cell type based on gene expression. This step was performed manually by producing a dot plot to compare expression of key marker genes in the single-cell profiles of mouse skin cells across clusters [18]. Clusters that did not perfectly match these expression patterns were annotated using CellMarker 2.0’s Cell Annotation tool [19].

Differential expression (DE) was run on each cell type using Seurat’s FindMarkers command using the Model-based Analysis of Single-cell Transcriptomes (MAST) v1.28.0 test [14,20]. This command was run on each cell type in the Seurat object, ensuring that each cell had its own expression profile. Samples from wild-type mice were set as the control, and samples from knock-in mice were set as the comparison condition. All genes meeting expression-quality criteria were tested, with minimum expression percentage and log2 fold-change filters set to 0 during model fitting to avoid excluding genes prior to statistical testing (Table S2). Gene-level *p*-values were corrected for multiple comparisons using the multiple-testing adjustment implemented by Seurat, and genes with an absolute log2 fold change ≥ 0.3 and adjusted *p* ≤ 0.05 were considered differentially expressed (Table S3). For downstream pathway prioritization and visualization, a more stringent subset of genes with an absolute log2 fold change ≥ 1.0 and adjusted *p* ≤ 1 × 10^−20^ was used. Functional enrichment analysis of the prioritized fibroblast DEG set was performed using iPathwayGuide^TM^ (AdvaitaBio Corporation, https://ipathwayguide.advaitabio.com/ accessed 4 November 2025) [21,22]. Statistical significance for pathway and Gene Ontology enrichment was based on the multiple-testing-adjusted *p*-values reported by the platform. The CellChat tool was used to infer ligand–receptor-mediated communication networks from gene-expression data. Because the CellChat tool outputs represent computationally inferred interaction probabilities rather than direct biological measurements, these analyses were interpreted as hypothesis-generating and are reported as inferred changes in intercellular communication rather than as experimentally validated signaling events. Functional analysis of the most important genes in Fibroblast cells was run using Changes in cell–cell interactions and were characterized using the CellChat tool [23]. Single-cell datasets have been uploaded to GEO with an accession number of GSE343646 and a scheduled public release date of 22 August 2026.

## 3. Results

### 3.1. Adamts2 Is Broadly Expressed in Developing and Adult Connective Tissue Compartments

To define the developmental and tissue distribution of *Adamts2*, we examined mouse embryonic in situ hybridization data together with human bulk and single-cell transcriptomic datasets (Figures S1–S4). At E14.5, *Adamts2* transcripts were broadly detected throughout the developing mouse embryo, with expression concentrated in connective tissue-rich and mesenchymal compartments. Prominent signal was evident in the developing dermis and subcutaneous tissues, craniofacial mesenchyme, tissues surrounding the vertebral column and cartilaginous skeletal elements, and mesenchymal regions of developing thoracic and abdominal organs (Figure S1). Analysis of adult human tissues similarly demonstrated widespread but variable *ADAMTS2* mRNA expression. Bulk data showed the highest expression in cultured fibroblasts, with substantial expression also present in adipose tissue, arteries, lung, breast, uterus, vagina, and multiple gastrointestinal and musculoskeletal tissues (Figure S2). By comparison, *ADAMTS2* expression was relatively low across most brain regions and in EBV-transformed lymphocytes. Expression patterns were broadly comparable between female and male samples. Single-cell analysis further localized *ADAMTS2* mRNA expression predominantly to fibroblasts and related mesenchymal populations. Across multiple organs, expression was enriched in fibroblasts, myofibroblasts, smooth muscle cells, pericytes, and selected stromal or mesenchymal progenitor populations, whereas most immune and neural cell populations showed little or inconsistent expression (Figure S2). Exon-level and splice-junction analyses demonstrated coordinated expression across the *ADAMTS2* gene. Cultured fibroblasts exhibited the strongest and most continuous coverage across the canonical exons, while lower but broadly concordant exon usage was observed in connective tissue-rich organs, including arterial, adipose, reproductive, pulmonary, and visceral tissues (Figure S3A). Sequential exon–exon junctions were also consistently detected in these tissues and generally paralleled total transcript abundance (Figure S3B). Together, the concordant exon and junction profiles support predominant expression of a full-length *ADAMTS2* transcript rather than extensive tissue-specific exon skipping. Collectively, these data demonstrate that *ADAMTS2* is expressed broadly from embryonic development through adulthood and is preferentially associated with fibroblasts and other mesenchymal cell populations in connective tissue-rich organs.

### 3.2. Gross Phenotype Observations and Loss of Function Validation of Adamts2^Q226*^ In Vivo

To investigate the functional consequences of the human disease-causing variant, we generated a knock-in mouse carrying the corresponding pathogenic mutation associated with dEDS (Figure S4). Homozygous Adamts2^Q226*/Q226*^ mice developed a severe and fully penetrant connective-tissue fragility phenotype and were readily distinguishable from control littermates by pronounced skin laxity, spontaneous bruising, and skin tearing during routine handling (Figure S5). Gross examination revealed large, full-thickness wounds over the dorsal shoulder region that arose spontaneously or following minimal mechanical stress, accompanied by marked skin redundancy, loss of dermal integrity, and ulcerative dermatitis (Figure S5A). This fragility was particularly apparent during cervical dislocation, when the skin separated from the underlying tissues and could be displaced along the body and tail with minimal resistance (Figure S5B). Reflection of the skin exposed extensive subcutaneous hemorrhage beneath areas that appeared relatively intact externally, consistent with combined fragility of the dermal ECM and small blood vessels (Figure S5C). Abdominal examination also demonstrated substantial internal hemorrhage, including blood accumulation surrounding the abdominal organs and evidence of vascular or soft-tissue rupture (Figure S5D). Heterozygous Adamts2^Q226*/+^ mice appeared grossly normal, consistent with the autosomal recessive inheritance of dEDS in humans.

**Figure 1:**
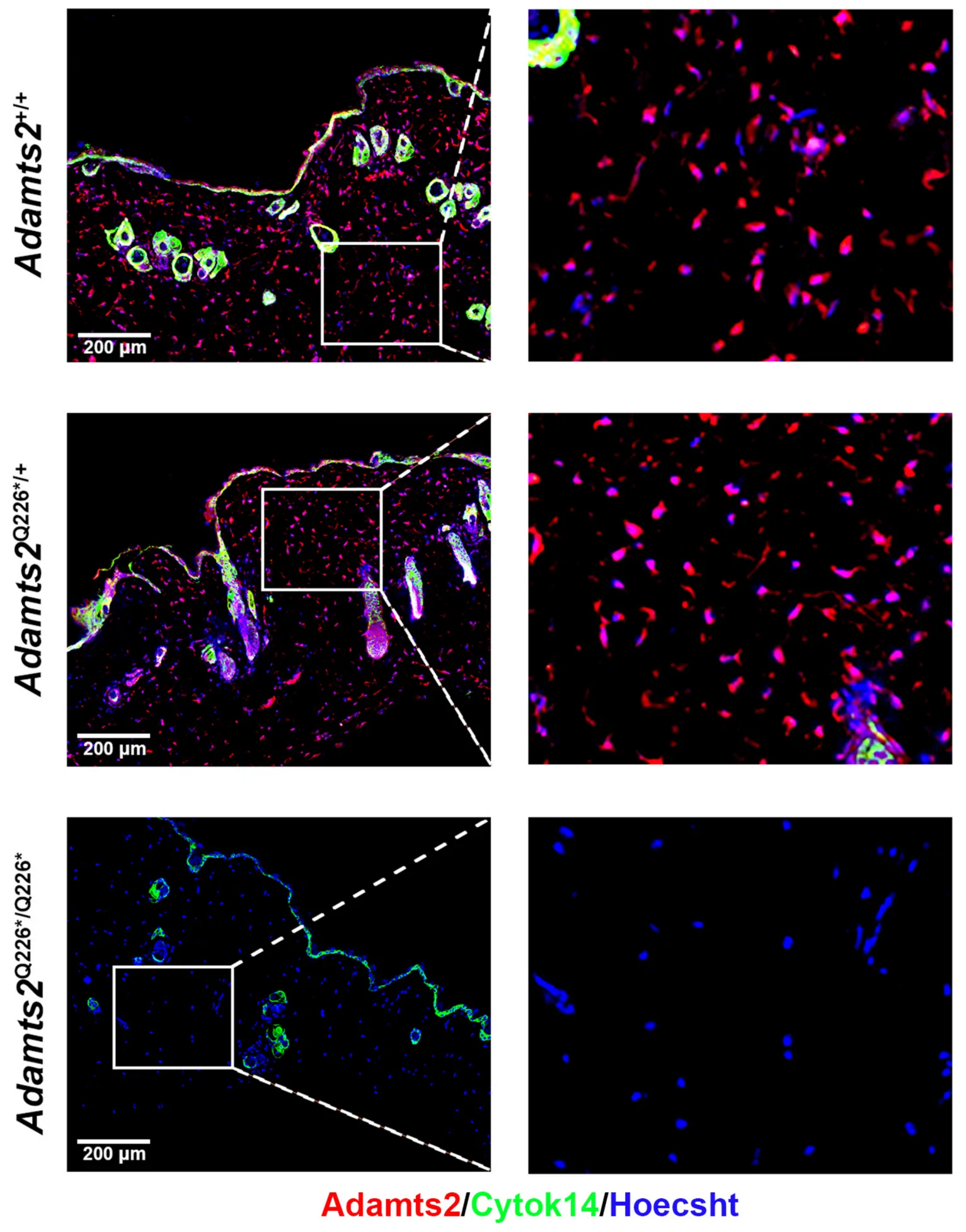
Loss of ADAMTS2 expression in Adamts2^Q226*/Q226*^ mouse skin. Representative immunofluorescence images of dorsal skin from *Adamts2^+/+^*, heterozygous Adamts2^Q226*/+^, and homozygous Adamts2^Q226*/Q226*^ mice (*n* = 4 per genotype) stained for Cytokeratin 14 (green), ADAMTS2 (red), and Hoechst (blue). In wild-type and heterozygous skin, Adamts2 is readily detected in the dermis underlying a continuous Cytok14-positive epidermis, whereas Adamts2 is undetected in homozygous knock-in mutants. Scale bar, 200 μm.

**Figure 2:**
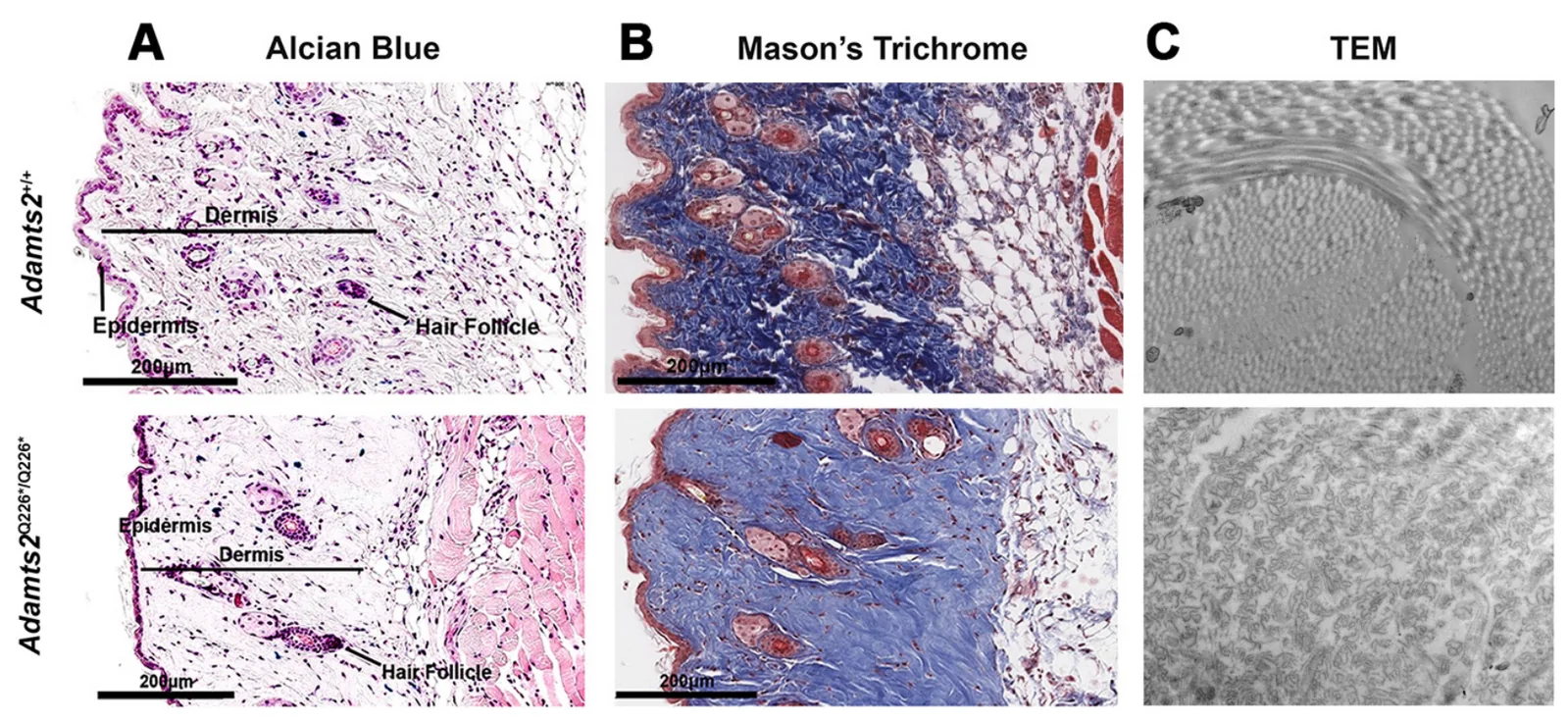
Histological and ultrastructural evidence of dermal ECM disorganization in homozygous Adamts2^Q226*/Q226*^ mice. (**A**,**B**) Representative Alcian blue and Masson’s trichrome staining of dorsal skin from *Adamts2^+/+^* (*n* = 3) and homozygous Adamts2^Q226*/Q226*^ mice (*n* = 6) showing loss of normal dermal architecture and increased ECM disorganization in homozygous mutants compared with wild-type and heterozygotes. Scale bars, 200 μm. (**C**) Transmission electron microscopy (TEM) images of dermal collagen fibrils from *Adamts2^+/+^* and homozygous Adamts2^Q226*/Q226*^ mice demonstrating tightly packed, uniformly cross-banded fibrils in wild-type skin and irregular, hieroglyphic fibril profiles characteristic of Dermatosparaxis in homozygous mutants.

Given the established role of ADAMTS2 in dermal connective tissue and the prominent skin phenotype of homozygous Adamts2^Q226*/Q226*^ mice, we examined ADAMTS2 protein expression in dorsal skin as well as potential histological phenotypes. Immunofluorescence revealed abundant dermal ADAMTS2 staining in wild-type (*Adamts2^+/+^*) mice, reduced signal in heterozygous littermates (Adamts2^Q226*/+^), and near-complete loss in homozygous knock-in mutants (Adamts2^Q226*/Q226*^), while the Cytokeratin 14-positive epidermis remained continuous across genotypes (*n* = 4 per genotype; Figure 1). Alcian blue and Masson’s trichrome staining of dorsal skin showed normal dermal architecture in wild-type mice, while homozygous Adamts2^Q226*/Q226*^ animals had disrupted collagen fiber alignment, expanded interfibrillar spaces, and a less distinct dermal–epidermal junction (*n* = 4 per genotype; Figure 2A,B). Transmission electron microscopy (TEM) of dorsal skin showed tightly packed, uniformly cross-banded collagen fibrils in controls versus irregular, variably sized “hieroglyphic” fibrils in homozygous Adamts2^Q226*/Q226*^ mutants (Figure 2C).

### 3.3. Collagen I Deposition and Processing Are Impaired in Mutants

**Figure 3:**
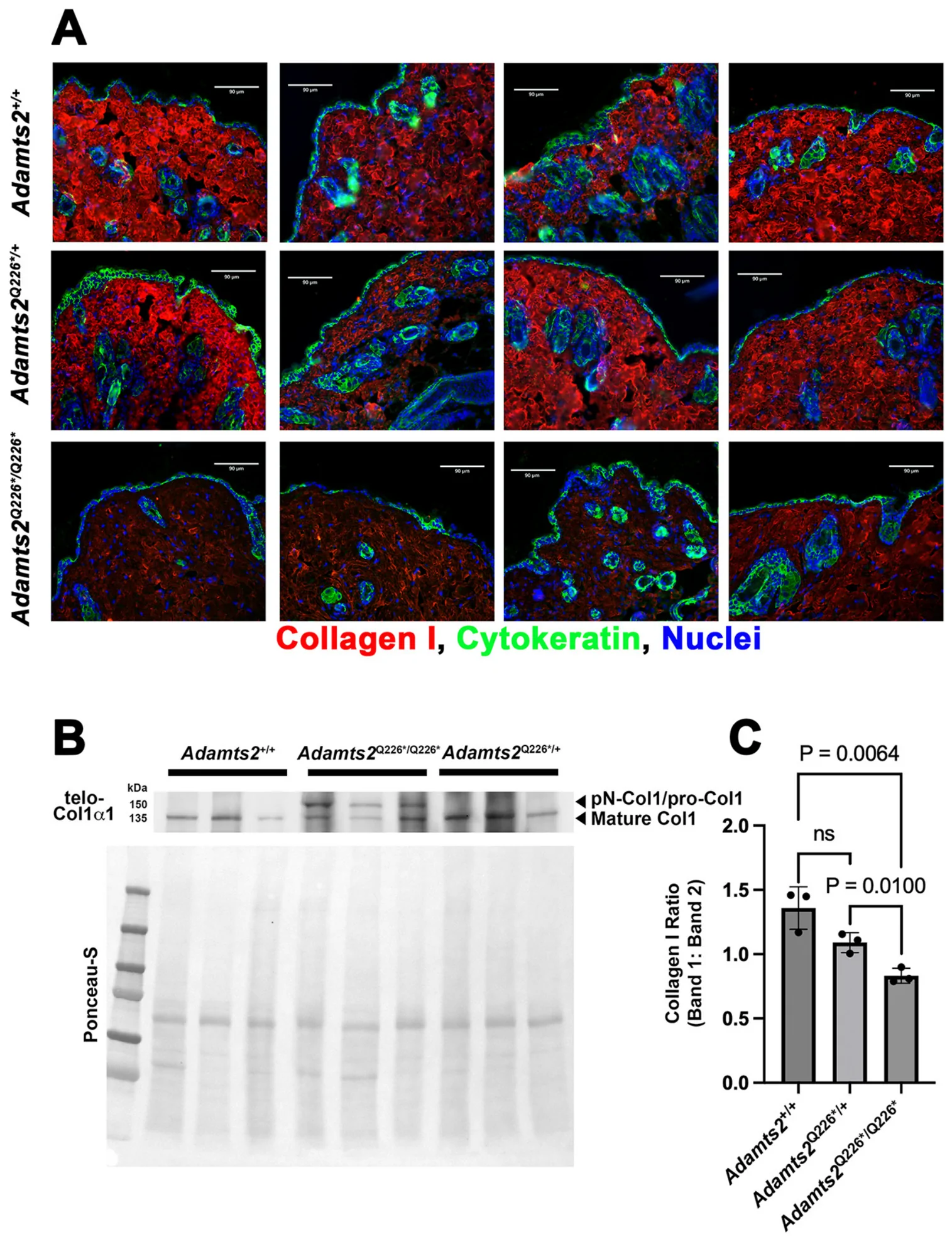
Collagen I deposition and processing are impaired in homozygous Adamts2^Q226*/Q226*^ mice. (**A**) Immunofluorescence staining of dorsal skin from *Adamts2^+/+^*, heterozygous Adamts2^Q226*/+^, and homozygous Adamts2^Q226*/Q226*^ mice (*n* = 4 per genotype) showing reduced type I Collagen (Col I, red) in the dermis of homozygous mice, with Cytokeratin 14 (Cytok14, green) marking the epidermis and Hoechst (nuclei-blue) labeling nuclei. Scale bars, 200 μm. (**B**) Western blot of skin lysate (*n* = 3 per genotype) probed for Collagen I, demonstrating increased procollagen I and reduced processed collagen in homozygous Adamts2^Q226*/Q226*^ mice compared with wild-type and heterozygous littermates. Ponceau staining is shown as a loading control. (**C**) Quantification of the processed-to-unprocessed Collagen I ratio with data representing independent biological samples (*n* = 3 mice per genotype) and are shown as mean ± SEM. Statistical significance was assessed by one-way ANOVA followed by Tukey’s multiple-comparisons test. *p*-values are shown for individual comparisons. Quantification of the ratio of processed to unprocessed Collagen I reveals a significant decrease in homozygous mutants relative to wild-type (*p* = 0.0064) and heterozygous (*p* = 0.0100) mice.

Immunofluorescent staining for type I collagen (red) demonstrated a marked change in homozygous Adamts2^Q226*/Q226*^ mice compared with wild-type and heterozygous littermates (*n* = 4 per genotype; Figure 3A). Collagen I staining appears more abundant and broadly distributed in the wild-type and Adamts2^Q226*/+^ heterozygous skin, consistent with a denser collagen-rich matrix. In contrast, the homozygous Adamts2^Q226*/Q226*^ skin shows a more heterogeneous staining, with areas of decreased signal and less continuous collagen organization, suggesting altered collagen deposition and/or matrix architecture. Although slight changes in collagen I expression may be apparent between wild-type and heterozygous *Adamts2* skin, the differences between wild-type and homozygous are more profound, thereby supporting a recessive effect of the Adamts2^Q226*^ allele on collagen deposition at the tissue level. As the collagen differences could potentially be explained by altered collagen processing, Western analyses of soluble skin extracts were performed (Figure 3B,C). These Western blot analyses (*n* = 3 per genotype) revealed accumulation of procollagen I and reduced levels of processed collagen in homozygous mutants relative to both wild-type and heterozygous animals (Figure 3B). Densitometric quantification confirmed a significant decrease in the ratio of processed to unprocessed Collagen I in homozygous Adamts2^Q226*/Q226*^ mice compared with wild-type (*p* = 0.0064) and heterozygous (*p* = 0.0100) groups, consistent with impaired N-terminal propeptide removal in the absence of functional ADAMTS2 protein (Figure 3C). For transparency, we have provided the full, uncropped, raw image from the Western blot in Figure S6.

### 3.4. Digital Pathology Identifies Adamts2-Dependent Alterations in Tissue Architecture

**Figure 4:**
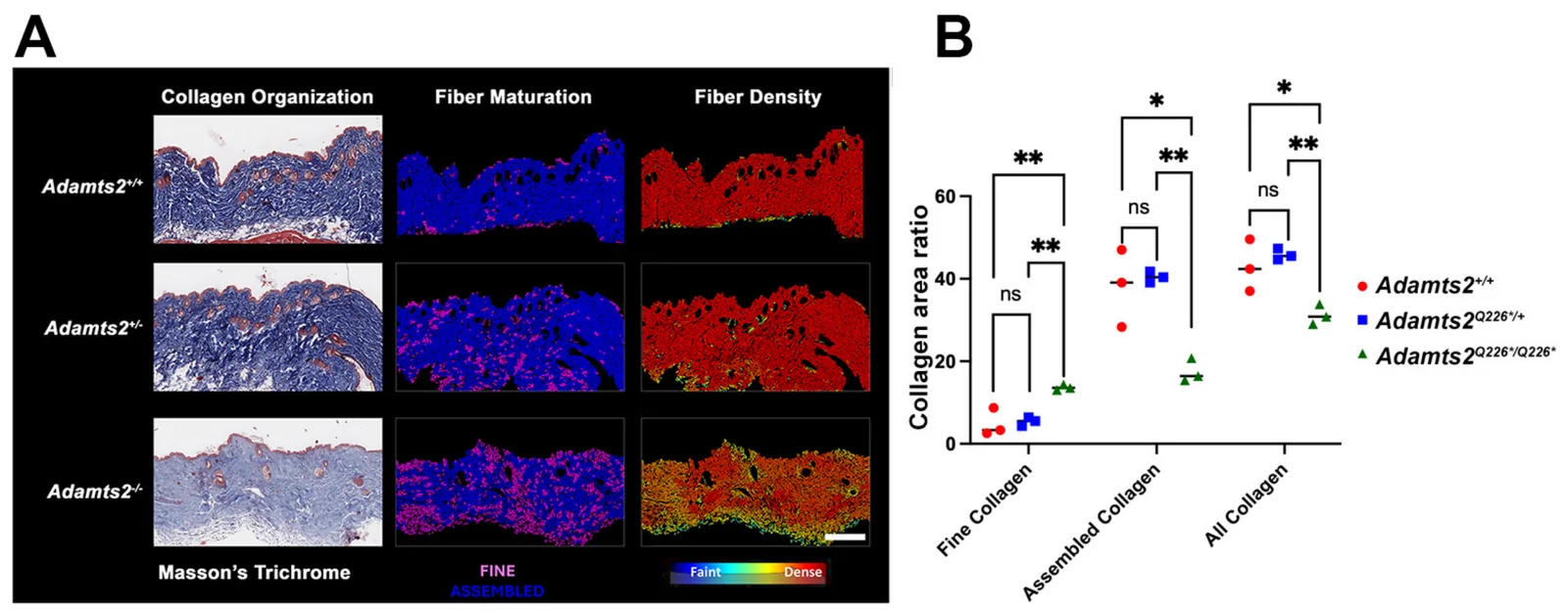
ADAMTS2 deficiency disrupts dermal collagen organization and alters collagen fiber maturation and density. (**A**) Representative Masson’s trichrome-stained skin sections from *Adamts2*^+/+^, heterozygous *Adamts2*^Q226*/+^, and homozygous *Adamts2*^Q226*/Q226*^ mice, shown alongside digital image-analysis maps of collagen organization, fiber maturation, and fiber density. Collagen organization maps highlight differences in dermal architecture and fiber alignment. Fiber maturation maps distinguish fine/immature (pink) collagen fibers from assembled/mature fibers (blue), whereas density maps display collagen distribution from faint to densely stained regions. Homozygous mutant skin exhibited marked disruption of collagen architecture, reduced mature/assembled collagen, and an increased proportion of densely stained but structurally disorganized collagen compared with wild-type skin. Heterozygous skin showed an intermediate phenotype, albeit not statistically significant. (**B**) Quantification of fine collagen, assembled collagen, and total collagen content across genotypes. No significant differences were observed between control and heterozygous knock-in mice. In contrast, homozygous knock-in mice differed significantly from controls for fine (*p* = 0.0048), assembled (*p* = 0.0107), and total collagen (*p* = 0.0256). Homozygous knock-in mice also differed significantly from heterozygotes for fine (*p* = 0.0067), assembled (*p* = 0.0065), and total collagen (*p* = 0.0097). * *p* < 0.05, ** *p* < 0.01.

To obtain a more in-depth and agnostic evaluation of the potential effect of ADAMTS2 on the regulation of collagen, quantitative fibrosis profiling of Masson’s Trichrome-stained dorsal skin revealed genotype-dependent alterations in dermal collagen architecture across the *Adamts2* allelic series (*n* = 3/genotype). *Adamts2^+/+^* skin showed dense, uniformly organized collagen with predominantly assembled fibers and high fiber density (Figure 4A). Heterozygous Adamts2^Q226*/+^ skin displayed an intermediate phenotype, with greater variability in fiber size, orientation, and density and an increased contribution of finer collagen structures. In contrast, homozygous Adamts2^Q226*/Q226*^ skin exhibited markedly reduced collagen density and organization, with diffuse, poorly bundled fibers, increased fine collagen structures, and fewer regions of densely packed matrix. Together, these findings indicate progressive impairment of collagen maturation and dermal architecture with increasing *Adamts2* allelic disruption.

### 3.5. ADAMTS2 Deficiency Disrupts Dermal Collagen Bulk, Maturation, and Architecture

**Figure 5:**
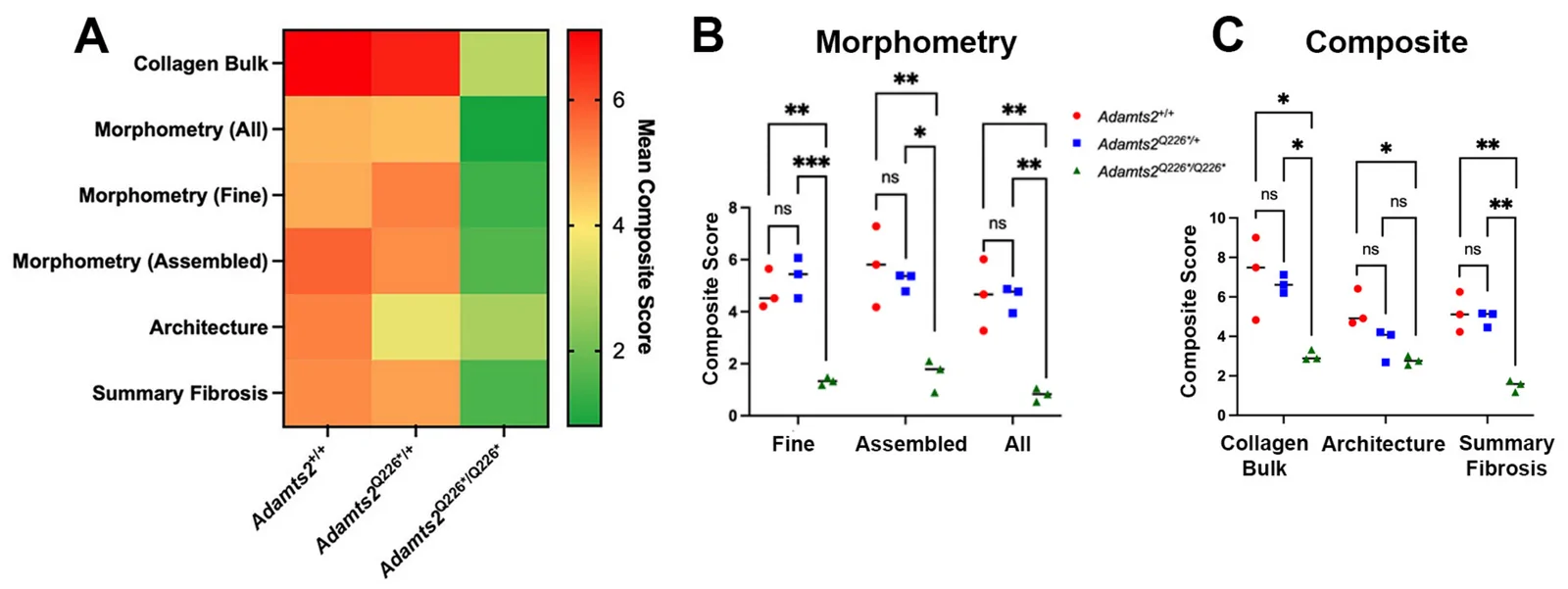
Composite histomorphometric analysis demonstrates broad reductions in dermal collagen quality and architecture following ADAMTS2 loss. (**A**) Heatmap showing mean composite scores for collagen bulk, overall morphometry, fine-fiber morphometry, assembled-fiber morphometry, collagen architecture, and summary fibrosis in skin from *Adamts2*^+/+^, heterozygous *Adamts2*^Q226*/+^, and homozygous *Adamts2*^Q226*/Q226*^ mice. Higher composite scores are shown in red and lower scores in green. Homozygous mutant skin displayed consistently reduced scores across all measured categories, whereas heterozygous skin generally showed intermediate or near-wild-type values. (**B**) Quantification of composite morphometry scores for fine collagen, assembled collagen, and all collagen fibers. Homozygous *Adamts2*^Q226*/Q226*^ skin exhibited significantly reduced morphometric scores compared with wild-type (*p* = 0.0013, *p* = 0.0050, and *p* = 0.0037) and heterozygous (*p* = 0.0006, *p* = 0.0102, *p* = 0.0044) for fine, assembled, and all tissues, respectively. Differences between wild-type and heterozygous groups were not significant. (**C**) Quantification of composite scores for collagen bulk, tissue architecture, and summary fibrosis. Homozygous mutant skin showed significant reductions in collagen bulk (*p* = 0.0172), architectural organization (*p* = 0.0129), and the overall fibrosis-associated composite score (*p* = 0.0011) relative to wild-type controls. A similar result was obtained when comparing potential differences between control and heterozygote Adamts2^Q226*/+^ with *p* = 0.0289 and 0.0017 for collagen bulk and fibrosis composite scores, respectively. No significant differences were observed between control and heterozygous *Adamts2^Q226/+^*, although there was a near-significant trend in the overall fibrosis architecture score (*p* = 0.07199. ns, not significant; * *p* < 0.05, ** *p* < 0.01, *** *p* < 0.001.

Composite digital pathology analysis demonstrated a genotype-dependent disruption of dermal organization across the *Adamts2* allelic series. *Adamts2^+/+^* skin showed the highest scores for collagen bulk, morphometry, tissue architecture, and overall fibrosis. Heterozygous Adamts2^Q226*/+^ samples were generally intermediate, and homozygous Adamts2^Q226*/Q226*^ tissue showed the lowest values (Figure 5A). Quantitative analyses confirmed significant reductions in fine and assembled collagen morphometry, total morphometry, collagen bulk, tissue architecture, and summary fibrosis in homozygous mutants compared with wild-type and/or heterozygous samples (Figure 5B,C). Heterozygous tissue was generally not significantly different from controls. Together, these findings indicate that biallelic *Adamts2* disruption causes a severe dermal ECM defect characterized by reduced collagen content, impaired fiber maturation, and loss of normal tissue organization. Full qFT and composite values are provided in Table S1.

### 3.6. Single-Nuclei Transcriptomics Reveal Fibroblast-Enriched ECM Disruption

**Figure 6:**
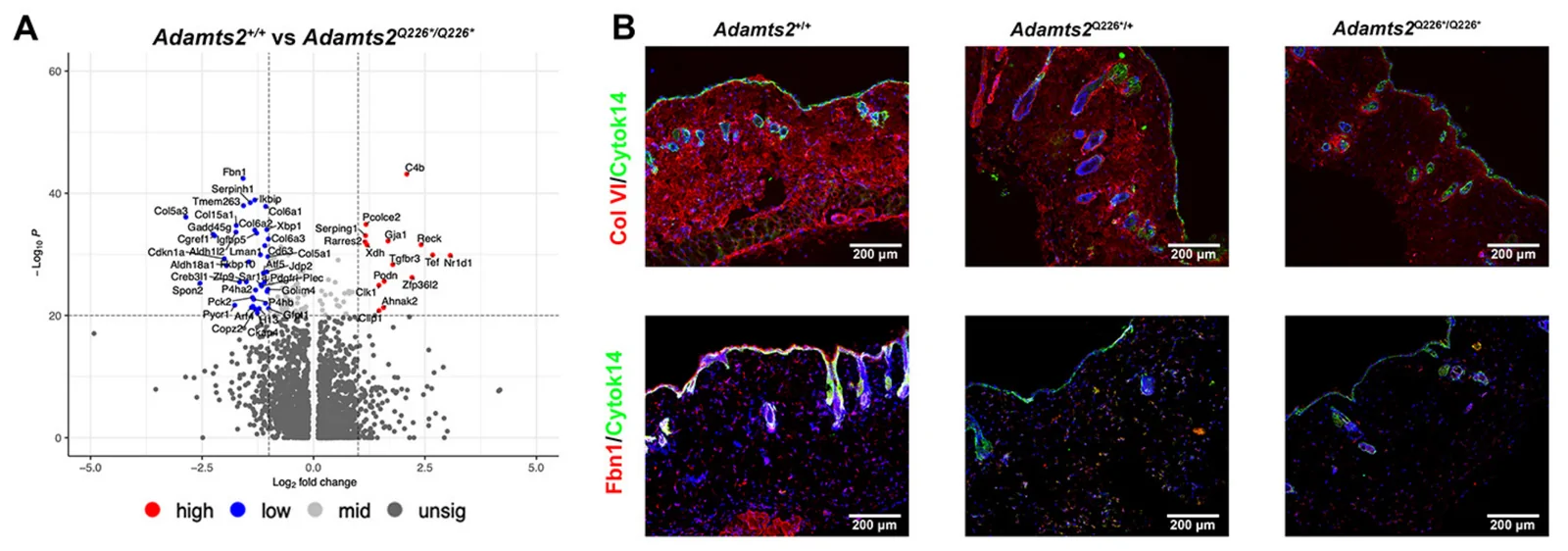
Single-nucleus RNA sequencing reveals fibroblast-enriched ECM dysregulation in homozygous Adamts2^Q226*/Q226*^ skin. (**A**) Volcano plot of differentially expressed genes (DEGs) in fibroblasts isolated from dorsal skin of wild-type *Adamts2^+/+^* and homozygous Adamts2^Q226*/Q226*^ mice, showing significant downregulation of multiple ECM and adhesion-related genes in homozygous Adamts2^Q226*/Q226*^ fibroblasts (FDR and fold-change thresholds as indicated). (**B**) Immunofluorescence staining of dorsal skin from *Adamts2^+/+^*, heterozygous Adamts2^Q226*/+^, and homozygous Adamts2^Q226*/Q226*^ mice (*n* = 4 per genotype) showing reduced type VI collagen (Col VI, red) and Fibrillin-1 (Fbn1, red) in the dermis of homozygous mice, with Cytokeratin 14 (Cytok14, green) marking the epidermis and Hoechst (nuclei-blue) labeling nuclei. Scale bars, 200 μm.

Single-nuclei RNA sequencing of FFPE dorsal skin was performed on *Adamts2^+/+^* and homozygous Adamts2^Q226*/Q226*^ mice (*n* = 4 per genotype). Clustering and cell type annotation identified major dermal and epidermal populations, including fibroblasts, keratinocytes, endothelial cells, immune cells, and other stromal subtypes (Figure S7). Differential expression analysis across cell types showed that fibroblasts exhibited the largest number and magnitude of transcriptional changes in homozygous Adamts2^Q226*/Q226*^ mice relative to controls, whereas non-fibroblast populations demonstrated modest transcriptional shifts (Figure S8 and Figure 6A). In fibroblasts, ECM genes were significantly downregulated, including several collagens, fibrillin-1, and growth factor genes (Figure 6A). Consistent with these transcriptomic findings, immunohistochemistry demonstrated reduced dermal staining for Collagen VI (Col VI) and Fibrillin-1 (Fbn1) in homozygous Adamts2^Q226*/Q226*^ skin compared with controls. Adamts2^Q226*/+^ heterozygote skin showed a qualitative intermediate staining pattern, suggesting a partial reduction in protein abundance (Figure 6B). Together, these findings support that at least some of the transcript-level changes observed in the snRNAseq translate to protein changes in homozygous Adamts2^Q226*/Q226*^ mice.

### 3.7. Functional Enrichment and CellChat Analyses Identify Coordinated Disruption of ECM Programs and Intercellular Communication in Homozygous Adamts2^Q226*/Q226*^ Skin

**Figure 7:**
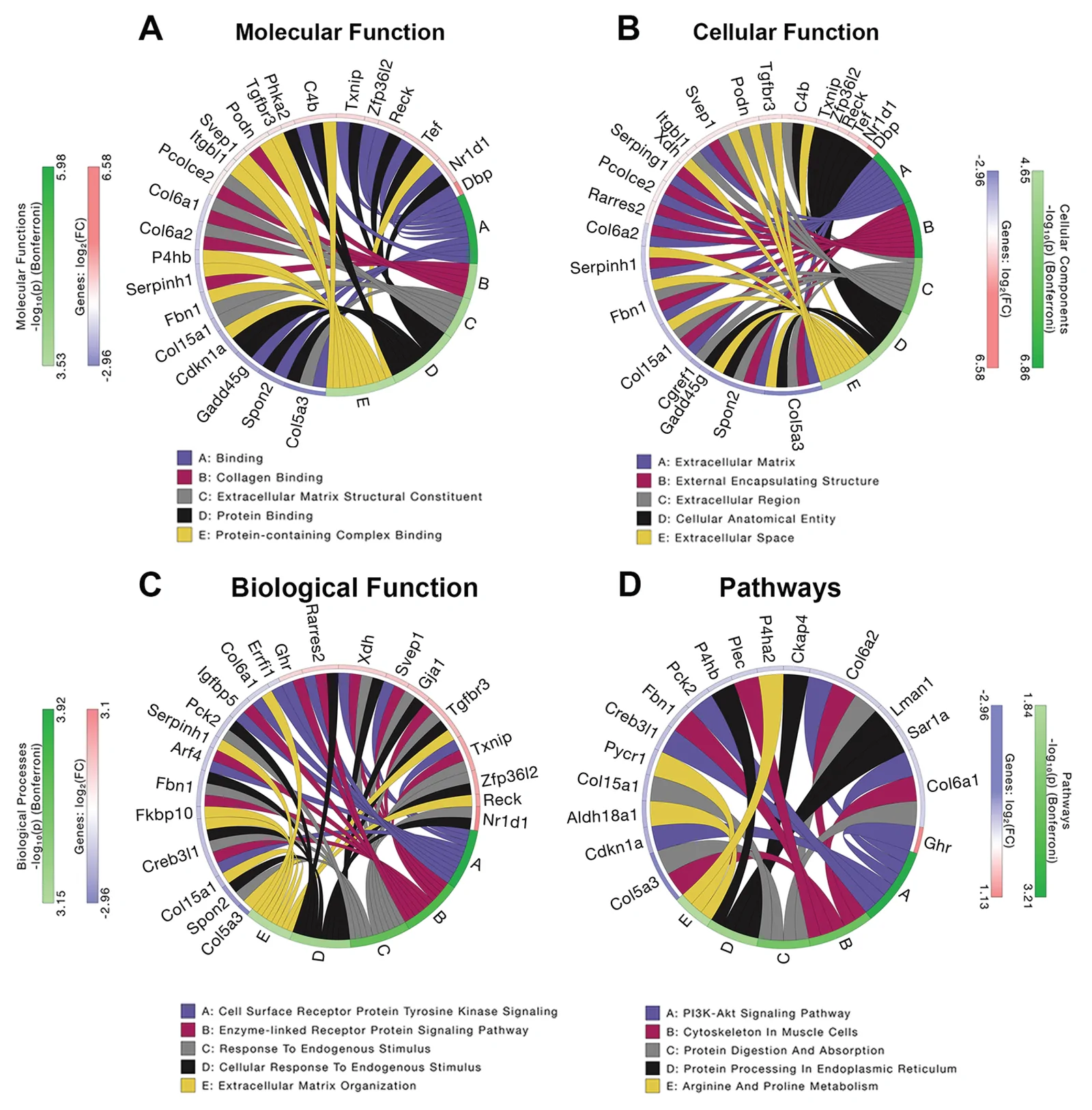
Circos plots summarizing functional enrichment of differentially expressed genes in homozygous Adamts2^Q226*/Q226*^ skin compared to controls. Enriched categories are grouped according to (**A**) molecular functions, (**B**) cellular components, (**C**) intracellular signaling pathways, and (**D**) biological processes. Individual genes are connected to their associated functional annotations, highlighting prominent changes in ECM structure and organization, protein and growth factor binding, cell-surface receptor signaling, cytoskeletal regulation, integrin-associated signaling, responses to endogenous stimuli, and extracellular matrix remodeling. Color scales indicate gene-level fold change and enrichment significance.

To further define the molecular programs altered in *Adamts2* mutant fibroblasts, DEGs were analyzed by pathway analyses and gene ontology enrichment tools. Consistent with the observed downregulation of ECM-associated transcripts, molecular function enrichment identified significant changes in categories related to collagen binding and extracellular matrix structural constituent activity (Figure 7A). These enriched terms were driven by multiple ECM and matrix-associated genes, including *Col6a1*, *Col6a2*, *Col15a1*, *Col5a3*, *Fbn1*, *Spon2*, *Pcolce2*, *Svep1*, *Podn*, and *Itgbl1*, supporting a broad disruption of matrix-related fibroblast programs. Cellular component analysis similarly demonstrated enrichment for genes associated with the ECM and extracellular space (Figure 7B). These findings indicate that the transcriptional consequences of *Adamts2* disruption are concentrated within genes encoding secreted, matrix-associated, and extracellular structural components. Several collagen and matrix assembly genes, including *Col6a2*, *Col15a1*, *Fbn1*, *Serpinh1*, *Pcolce2*, *Svep1*, and *Itgbl1*, contributed to these enriched categories, reinforcing the conclusion that *Adamts2* mutant fibroblasts exhibit impaired ECM organization. Pathway-level analysis further revealed enrichment of pathways associated with PI3K-Akt signaling, cytoskeletal regulation, protein digestion and absorption, protein processing in the endoplasmic reticulum, and arginine and proline metabolism (Figure 7C). The PI3K-Akt pathway inferred altered expression of ECM and receptor-associated genes, including *Col6a1*, *Col6a2*, *Col15a1*, *Fbn1*, *Creb3l1*, *Aldh18a1*, and *Pck2*, suggesting that matrix disruption is accompanied by changes in growth factor, survival, metabolic, and mechanotransduction-associated signaling networks. Mapping of differentially expressed genes onto the PI3K-Akt signaling pathway showed altered expression of upstream cytokine/growth factor and ECM-associated nodes, with additional downstream changes in genes linked to cell-cycle regulation and survival signaling. Biological process enrichment supported each of these findings, identifying significant enrichment of cell surface receptor protein tyrosine kinase signaling, enzyme-linked receptor protein signaling, response to endogenous stimulus, cellular response to endogenous stimulus, and ECM organization (Figure 7D). Together, these pathway analyses demonstrate that homozygous Adamts2^Q226*/Q226*^ fibroblasts show broad changes in ECM composition, collagen-associated matrix assembly, extracellular organization, and receptor-linked signaling pathways.

**Figure 8:**
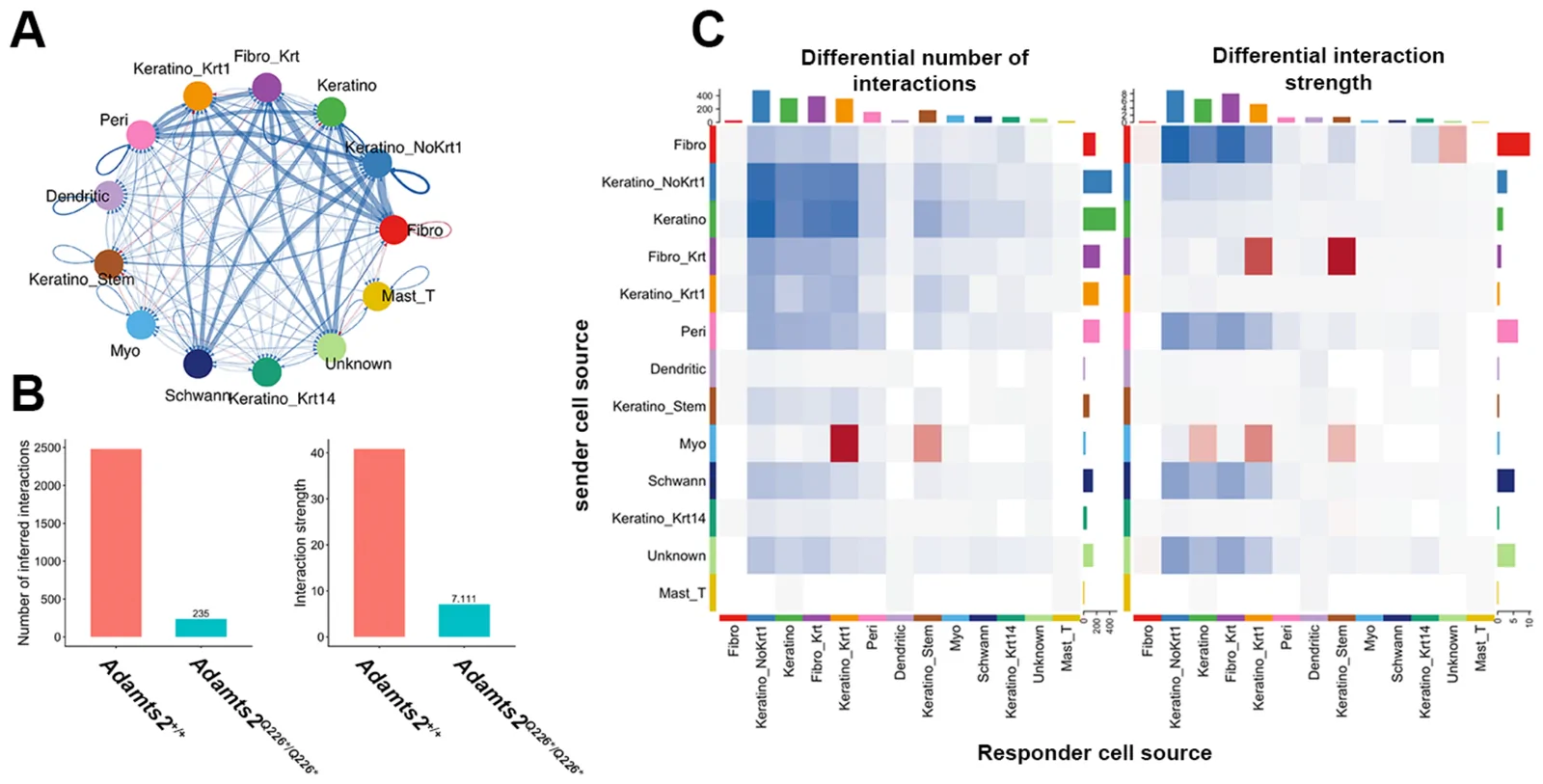
CellChat analyses of snRNAseq data from Adamts2^Q226*/Q226*^ and controls showing disrupted intercellular communication within the skin. (**A**) CellChat-derived network depicting predicted intercellular communication among major skin cell populations, including fibroblasts, keratinocyte subsets, pericytes, myocytes, Schwann cells, dendritic cells, mast cells, and cells of uncertain identity. Node size represents the relative abundance or communication contribution of each cell population, and connecting lines represent predicted ligand–receptor interactions; line thickness reflects interaction strength. (**B**) Comparison of the total predicted number of intercellular interactions and aggregate interaction strength between control and homozygous Adamts2^Q226*/Q226*^ skin. Homozygous mutant tissue exhibited a marked reduction in both the number and overall strength of predicted cell–cell interactions. (**C**) Heatmaps showing genotype-dependent differences in communication between sender and receiver cell populations, including differential number of predicted interactions and interaction strength. Blue shading indicates communication reduced in homozygous Adamts2^Q226*/Q226*^ skin relative to wild-type tissue, whereas red shading indicates increased communication. The most widespread losses involved communication between fibroblasts and multiple keratinocyte populations, as well as interactions involving pericytes, Schwann cells, myocytes, and other stromal or epithelial populations.

To determine whether these transcriptional changes were associated with altered cellular communication in skin tissue, CellChat analysis was performed on the snRNA-seq dataset. This analysis demonstrated a marked reduction in inferred cell–cell interactions in homozygous Adamts2^Q226*/Q226*^ samples compared with controls (Figure 8A,B). Differential interaction plots showed broad decreases in communication among multiple skin-resident cell populations, including fibroblast, keratinocyte, stromal, immune, Schwann, perivascular, dendritic, mast cell, and myeloid populations (Figure 8A). Quantitative CellChat summaries further showed that both the number of inferred interactions and the overall interaction strength were reduced in homozygous Adamts2^Q226*/Q226*^ skin (Figure 8C).

Differential heatmaps confirmed that these reductions were not confined to a single-cell population but reflected a broader loss of intercellular signaling across the tissue microenvironment. Deeper examination of the differential interaction matrices indicated that the most pronounced communication defects involved fibroblasts and keratinocyte-lineage populations (Figure 8C). Fibroblast signaling with non-Krt1 keratinocytes, conventional keratinocytes, and the fibroblast–keratinocyte transitional population showed some of the largest reductions in both the inferred number and strength of interactions. Communication among keratinocyte subtypes was also broadly diminished, particularly across the Keratino_NoKrt1, Keratino, Fibro_Krt, and Keratino_Krt1 populations, suggesting disruption of coordinated signaling across the dermal–epidermal interface. Perivascular cells also displayed substantial losses of interaction number and strength with fibroblasts and several keratinocyte populations. Schwann cells and the unclassified stromal population showed additional reductions in communication with fibroblast and epidermal cell types, indicating that the effects of ADAMTS2 deficiency extend beyond matrix-producing cells to vascular- and neural-associated compartments. In contrast, dendritic, mast-cell/T-cell, and myoid populations exhibited fewer or more selective changes, although isolated increases in specific interaction pairs were observed.

Overall, the strongest and most consistent losses centered on fibroblast–keratinocyte, keratinocyte–keratinocyte, and perivascular–epidermal communication. These findings suggest that *Adamts2* deficiency preferentially disrupts signaling networks in the skin responsible for maintaining dermal–epidermal organization, stromal support, and tissue homeostasis, rather than producing a uniform reduction across all cell populations.

## 4. Discussion

**Figure 9:**
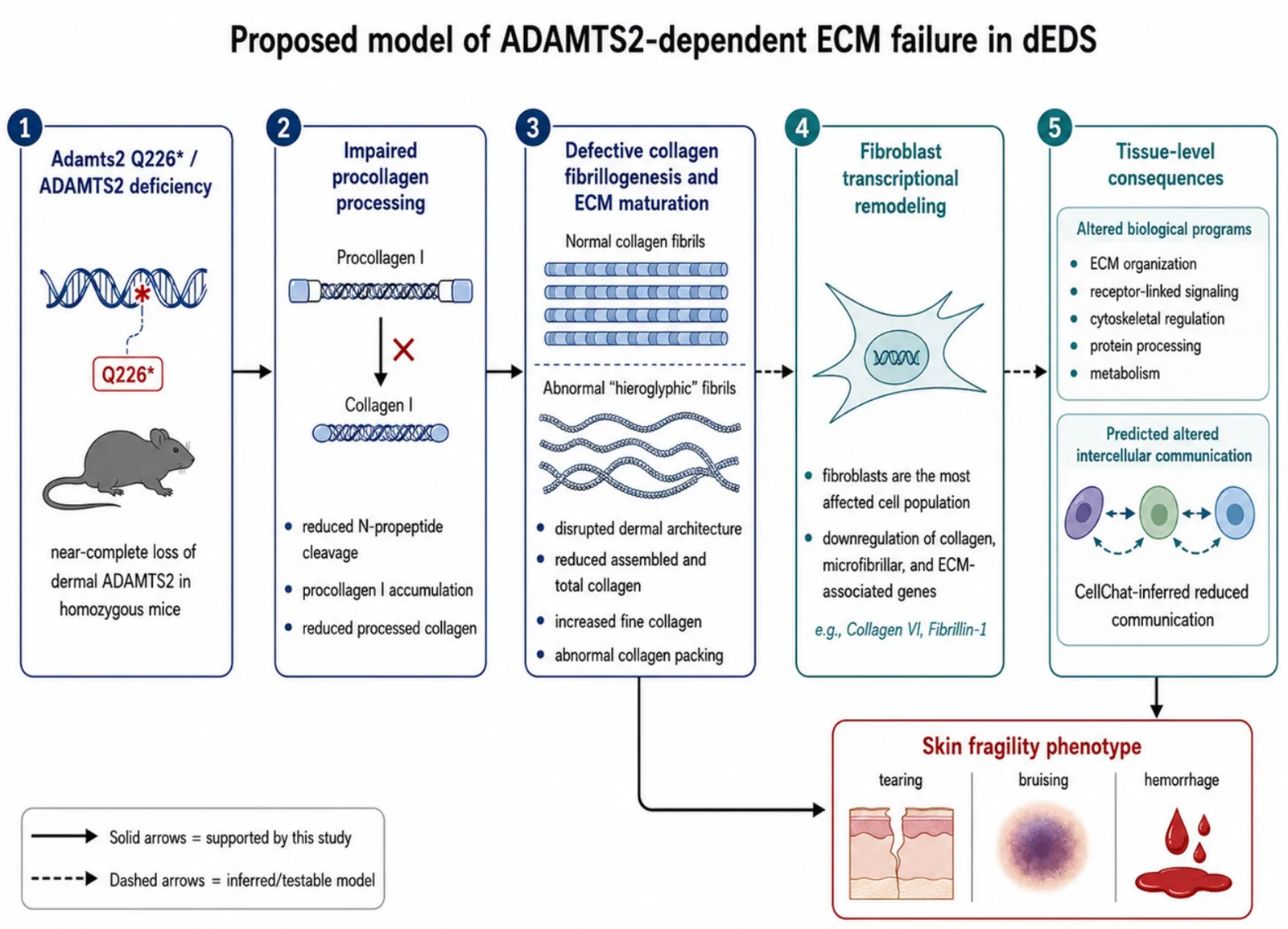
Proposed model of ADAMTS2-dependent ECM failure in dEDS. ADAMTS2 deficiency impairs procollagen processing, disrupts collagen fibrillogenesis and dermal ECM organization, and promotes fibroblast transcriptional remodeling and altered cell–cell communication, collectively contributing to skin fragility, bruising, and hemorrhage.

This study establishes the first patient-analog knock-in mouse model of dermatosparaxis Ehlers–Danlos syndrome (dEDS) carrying the homozygous Adamts2^Q226*/Q226*^ mutation and provides new mechanistic insight into how ADAMTS2 deficiency alters connective tissue structure and function. Consistent with prior studies of *Adamts2*-null mice, homozygous *Adamts2*^Q226*/Q226*^ knock-in animals exhibited markedly reduced dermal ADAMTS2 expression, impaired type I procollagen processing, disorganized dermal architecture, and abnormal hieroglyphic collagen fibrils characteristic of dEDS. However, the present findings extend beyond confirmation of the canonical collagen-processing defect. By integrating biochemical analyses, quantitative digital pathology, single-nucleus transcriptomics, pathway analysis, and inferred intercellular communication, our data support a broader model in which loss of ADAMTS2 initiates a fibroblast-enriched cascade involving defective collagen maturation, secondary suppression of ECM programs, altered intracellular signaling, and disruption of tissue-wide cellular communication. A schematic summarizing our experimental and inferred findings is represented in Figure 9.

Historically, dEDS has been understood primarily as a connective tissue disorder of procollagen N-terminal processing. ADAMTS2 cleaves the N-propeptide of fibrillar procollagen, and failure of this step prevents normal lateral association and assembly of collagen fibrils. Our biochemical and ultrastructural findings are consistent with this established mechanism. Homozygous Adamts2^Q226*/Q226*^ skin showed accumulation of unprocessed procollagen I, a reduced ratio of processed to unprocessed collagen, and irregular fibril profiles on transmission electron microscopy. These findings directly connect the Adamts2^Q226*^ variant to the defining molecular and ultrastructural features of human dEDS. Nonetheless, there is evidence of proper procollagen cleavage, indicating that either some residual activity is preserved in the context of the Adamts2^Q226*^ variant, or compensation from other *Adamts* family members occurs. Importantly, the knock-in approach preserves the endogenous genomic and regulatory context of *Adamts2*, providing a model that more closely reflects the consequences of a disease-associated allele than a complete gene deletion.

The digital pathology analyses further demonstrate that the effects of ADAMTS2 deficiency extend beyond abnormal fibril shape. Homozygous mutant skin showed reduced collagen bulk, diminished total and assembled collagen, increased fine collagen, and broad loss of mature collagen architecture. These results suggest that inefficient procollagen processing compromises not only fibril morphology but also the progressive assembly, bundling, and spatial organization of collagen throughout the dermis. The relative increase in fine collagen likely represents an accumulation of poorly matured, incompletely assembled, or structurally unstable collagen elements that fail to form larger organized bundles. Thus, the dEDS matrix defect appears to reflect both qualitative abnormalities in fibril structure and a quantitative failure to construct and maintain a mature collagen-rich network. This distinction is important because conventional histology may identify gross disorganization but cannot fully capture the multidimensional nature of matrix failure. The digital pathology platform used here quantified collagen density, morphometry, maturation, and architecture across the tissue and revealed genotype-dependent effects that were not uniformly apparent by visual inspection alone. These results support the use of computational pathology as a sensitive method for evaluating connective tissue disorders, particularly when disease effects involve subtle changes in matrix organization rather than complete loss of a structural protein. Quantitative matrix phenotyping may also provide useful outcome measures for future therapeutic studies aimed at restoring collagen maturation or stabilizing tissue architecture.

A major finding of this study is that the consequences of ADAMTS2 deficiency are not restricted to extracellular collagen processing. Single-nucleus RNA sequencing identified fibroblasts as the cell population with the largest number and magnitude of transcriptional alterations in homozygous mutant skin. This is consistent with fibroblasts being the principal producers and organizers of the dermal ECM, but it also suggests that ADAMTS2 loss alters fibroblast state through a cell-autonomous or matrix-mediated feedback mechanism. The coordinated downregulation of multiple collagens, microfibrillar, and matrix-associated genes indicates that the Adamts2^Q226*^ variant does not simply leave procollagen uncleaved; it alters the broader transcriptional program required for matrix production, assembly, and maintenance. These findings support a model of maladaptive fibroblast feedback. Abnormal collagen processing may initially generate a structurally defective ECM, which then fails to provide normal mechanical and biochemical signals to resident fibroblasts. In response, fibroblasts may reduce expression of matrix components, alter cytoskeletal organization, and modify receptor-linked signaling pathways. This secondary response could amplify the original processing defect by further reducing production of proteins required for matrix integrity. In this way, a primary extracellular enzymatic defect may evolve into a broader disorder of fibroblast function and tissue homeostasis. The reduction in collagen VI and fibrillin-1 is particularly important because these proteins contribute to matrix compartments distinct from fibrillar type I collagen. Collagen VI is a major component of the pericellular matrix and helps connect cells to the surrounding interstitial collagen network. It contributes to cell adhesion, mechanical stability, and force transmission between fibroblasts and the extracellular matrix. Fibrillin-1 is essential for microfibril formation, elastic fiber organization, and the regulation of matrix-associated growth factors. Their coordinated reduction suggests that ADAMTS2 deficiency destabilizes multiple interconnected matrix systems rather than affecting type I collagen in isolation. Loss of collagen VI and fibrillin-1 could therefore impair pericellular organization, microfibril integrity, elastic recoil, and growth-factor availability, further increasing tissue fragility and compromising the ability of fibroblasts to sense and respond to their extracellular environment.

The pathway analyses reinforce the previously mentioned interpretations. Differentially expressed fibroblast genes were enriched in categories related to ECM organization, collagen binding, extracellular structural constituents, receptor tyrosine kinase signaling, PI3K–Akt signaling, cytoskeletal regulation, protein processing in the endoplasmic reticulum, and arginine and proline metabolism. These pathways can be understood as components of a coordinated response to abnormal matrix assembly. Altered PI3K–Akt and receptor tyrosine kinase signaling may reflect changes in growth-factor availability, integrin signaling, or matrix-dependent receptor activation. Changes in cytoskeletal pathways are consistent with disrupted cell–matrix attachment and altered mechanical loading. Enrichment of endoplasmic reticulum processing pathways may indicate altered handling of secreted matrix proteins or increased biosynthetic stress caused by defective procollagen maturation. Similarly, changes in arginine and proline metabolism may reflect altered collagen biosynthesis or an attempt to compensate for impaired matrix production.

Together, these pathway changes suggest that mutant fibroblasts exist within an abnormal mechanical and biosynthetic state. A less organized and less densely assembled collagen matrix would be expected to alter tissue stiffness, fiber topology, and the distribution of mechanical forces. Because fibroblasts depend on ECM cues to regulate cytoskeletal tension, transcription, survival, and matrix production, defective collagen assembly may produce a feed-forward loop in which altered matrix mechanics further suppress normal fibroblast function. Direct measurements of dermal stiffness, fibroblast traction forces, integrin signaling, and mechanosensitive transcriptional activity will be important for testing this model.

The CellChat analyses extend our findings, demonstrating that the number and overall strength of inferred interactions between cell types were markedly reduced in homozygous mutant skin. The largest and most consistent decreases involved fibroblast–keratinocyte, keratinocyte–keratinocyte, and perivascular–epidermal communication. Fibroblast signaling with conventional keratinocytes, non-Krt1 keratinocytes, and fibroblast–keratinocyte transitional populations was particularly affected, suggesting disruption of coordinated signaling across the dermal–epidermal interface. This pattern has several potential biological implications. Fibroblast–keratinocyte signaling contributes to epidermal differentiation, basement membrane maintenance, wound repair, and barrier homeostasis. Reduced communication between these compartments could therefore impair the ability of the epidermis and dermis to coordinate responses to mechanical stress or injury. Broad reductions among keratinocyte populations may further indicate altered epidermal maturation or reduced paracrine support within the epithelial compartment. These effects may help explain why a primary dermal matrix disorder produces skin manifestations that cannot be attributed solely to abnormal collagen fibril morphology. Perivascular cells also showed substantial reductions in communication with fibroblasts and keratinocyte populations. This finding raises the possibility that ADAMTS2 deficiency alters stromal support of the dermal microvasculature or impairs communication between vascular-associated cells and the surrounding matrix. Schwann cells and other stromal populations similarly demonstrated reduced interactions with fibroblast and epidermal cells, suggesting that the effects of matrix disorganization may extend into neural-associated and sensory compartments. The broad expression patterns throughout development and into adulthood support the hypothesis that phenotypes associated with this mouse, and the dEDS patients in general, likely extend significantly beyond the dermal manifestations. Although these relationships require direct validation, they support the concept that the ECM serves not only as structural support but also as a platform for organizing tissue-level signaling throughout the entire body.

dEDS is inherited as an autosomal recessive disorder, and heterozygous mice did not display the severe histologic, biochemical, or ultrastructural phenotype observed in homozygous mutants. However, selected quantitative matrix parameters and immunostaining patterns appeared intermediate. These observations suggest that partial reduction in ADAMTS2 activity may produce subtle changes in matrix architecture that are not readily detected by routine histology. This should not be interpreted as evidence that monoallelic ADAMTS2 variants cause dEDS; rather, it raises the possibility that the amount of functional ADAMTS2 influences collagen organization along a quantitative continuum. Larger studies incorporating biomechanical testing, ultrastructural analysis, aging, and mechanical challenge will be necessary to determine whether these subtle changes have functional significance.

The knock-in model described here offers several advantages for future investigation. Since we introduce a disease-associated variant while preserving the endogenous locus, this model provides a unique platform for examining mutation-specific effects, residual expression, nonsense-mediated decay, and potential therapeutic rescue. Comparison with complete *Adamts2* knockout models may help distinguish consequences of total gene absence from those produced by patient-relevant alleles. The model may also be useful for testing strategies directed at gene replacement, transcript rescue, restoration of procollagen processing, enhancement of compensatory N-proteinase activity, or stabilization of downstream matrix architecture.

## 5. Study Limitations

Several limitations should be considered when interpreting these findings. First, this study was designed primarily to establish and characterize the Adamts2^Q226*/Q226*^ knock-in model rather than to establish causal relationships among all downstream molecular changes. The prominent fibroblast transcriptional response identifies fibroblasts as the cell population most transcriptionally affected in the skin but does not establish that fibroblasts are the primary drivers of ECM failure. Similarly, pathway-enrichment analyses identify transcriptional programs associated with ADAMTS2 deficiency but do not directly demonstrate activation or inhibition of the corresponding signaling pathways. CellChat analyses infer potential ligand–receptor interactions from transcriptomic data and therefore should be considered predictions of altered intercellular communication rather than direct measurements of signaling. Future studies incorporating tissue biomechanics, fibroblast traction-force measurements, integrin/FAK and other mechanosensitive signaling assays, expanded protein-level validation, and genetic or pharmacologic rescue will be required to test the feed-forward mechanistic model proposed here. Second, the present characterization focused primarily on dorsal skin from relatively young mice. Although skin fragility is a defining feature of this condition, dEDS is a systemic connective-tissue disorder that can affect many tissue organ systems. The extensive hemorrhage observed in homozygous animals and broad developmental and adult expression of ADAMTS2 suggest that additional tissues may also be affected in these mice. Most studies included all three genotypes in the analyses, except for the TEM study. It remains possible that there are slight ultrastructural changes in the heterozygote knock-in mice that are not fully detectable from the alternative imaging approaches conducted in this report. Future studies should therefore evaluate this, as well as systematically evaluate vasculature, tendon, ligament, musculoskeletal, gastrointestinal, immune, and other connective-tissue-rich organs, and age- and sex-dependent phenotypes. The severe fragility and reduced viability of homozygous animals present practical limitations for various longitudinal studies.

## 6. Conclusions

In conclusion, the homozygous Adamts2^Q226*/Q226*^ knock-in mouse reproduces defining biochemical, histologic, and ultrastructural features of dEDS and provides a genetically precise model for investigating ADAMTS2-associated connective-tissue disease. The data demonstrate that defective procollagen processing is accompanied by profound alterations in collagen fibril maturation and dermal architecture, together with prominent fibroblast transcriptional remodeling and broad changes in ECM-associated gene programs. Computational analyses further predict alterations in signaling pathways and intercellular communication that provide testable hypotheses for future mechanistic studies. This model establishes a translational platform for defining mutation-specific disease biology and evaluating strategies aimed at restoring collagen processing and ECM homeostasis.

## Data Availability

The data supporting the findings of this study are available within the article and its Supplementary Materials. The complete quantitative digital-pathology dataset, including individual fibrosis traits and composite scores, is provided in Table S1. Raw and processed single-nucleus RNA-sequencing data generated in this study will be deposited in the NCBI Gene Expression Omnibus following publication. Publicly available datasets analyzed in this study were obtained from GenePaint (https://gp3.mpg.de/results/adamts2 accessed 1 June 2025), the Genotype-Tissue Expression project (https://gtexportal.org/home/ accessed 4 September 2025), and the Tabula Muris (https://figshare.com/projects/TabulaMurisTranscriptomiccharacterizationof20organs_and_tissues_from_Mus_musculus_at_single_cell_resolution/27733 accessed 4 March 2025) resources. Additional data are available from the corresponding author upon reasonable request.

## Supplementary Materials

The following supporting information can be downloaded at: https://www.mdpi.com/article/10.3390/biomedicines14091928/s1.

## Abbreviations

The following abbreviations are used in this manuscript:

ACS	Architecture Composite Score
ADAMTS2	A Disintegrin and Metalloproteinase with Thrombospondin Motifs 2
CCS	Collagen Composite Score
CRISPR	Clustered Regularly Interspaced Short Palindromic Repeats
DAPI	4′,6-Diamidino-2-phenylindole
DEG	Differentially Expressed Gene
dEDS	Dermatosparaxis Ehlers–Danlos Syndrome
ECM	Extracellular Matrix
EDS	Ehlers–Danlos Syndromes
FDR	False Discovery Rate
FFPE	Formalin-Fixed, Paraffin-Embedded
GEO	Gene Expression Omnibus
gRNA	Guide RNA
GTEx	Genotype-Tissue Expression
HRP	Horseradish Peroxidase
IACUC	Institutional Animal Care and Use Committee
LFC	Log2 Fold Change
MAST	Model-based Analysis of Single-cell Transcriptomics
MCS	Morphometric Composite Score
PBS	Phosphate-Buffered Saline
PCA	Principal Component Analysis
PCR	Polymerase Chain Reaction
Ph-FCS	Phenotype Fibrosis Composite Score
qFT	Quantitative Fibrosis Trait
RIPA	Radioimmunoprecipitation Assay
TEM	Transmission Electron Microscopy
UMAP	Uniform Manifold Approximation and Projection
WT	Wild Type
