# Supplementary material for: An Adamts2 Knock-In Model of Dermatosparaxis Ehlers–Danlos Syndrome Reveals Defective Collagen Maturation
Source: Biomedicines. 2026 Aug 27;14(9):1928. doi: 10.3390/biomedicines14091928 (PMC13604185; doi:10.3390/biomedicines14091928)
Supplement: Supplementary file 1 [file biomedicines-14-01928-s001.zip › Supplemental Figures and Tables 082126/Supplemental Figures Biomedicine 081326.pdf]

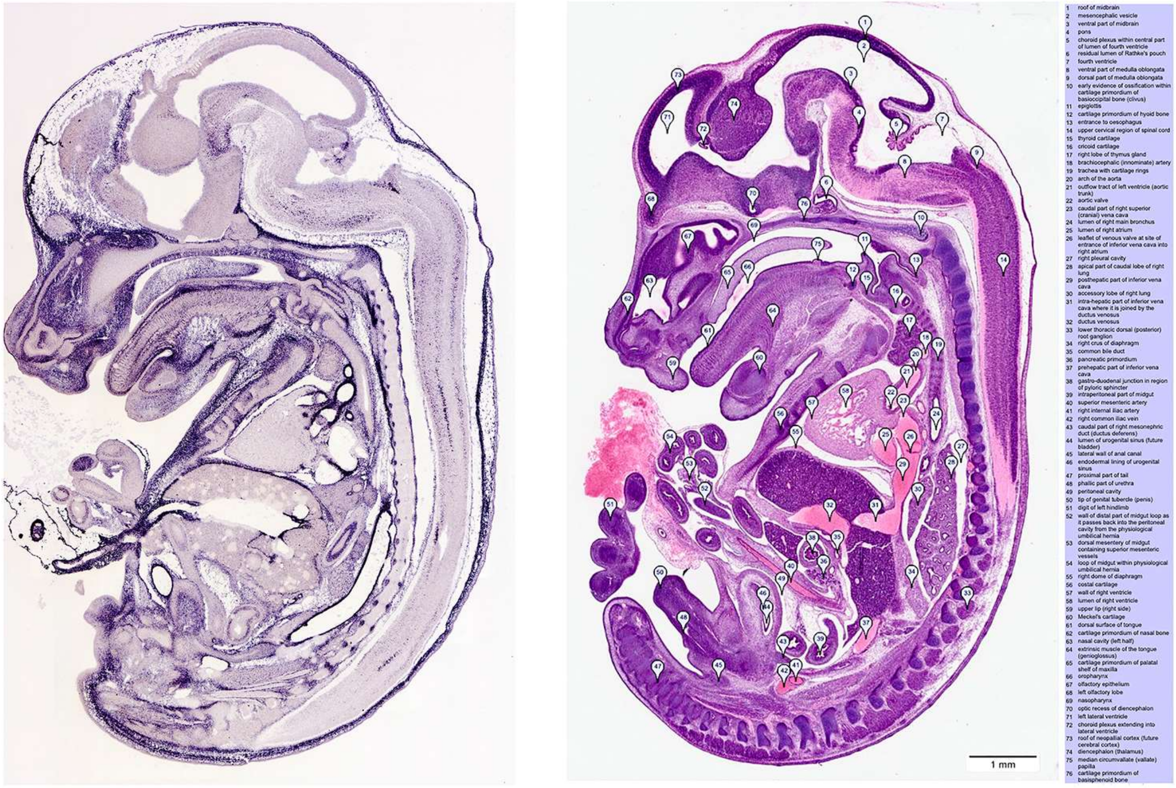

Figure S1. Developmental expression of *Adamts2* in the E14.5 mouse embryo. Representative sagittal RNA in situ hybridization image showing the distribution of *Adamts2* transcripts in an E14.5 mouse embryo (left), displayed alongside a stage-matched hematoxylin- and eosin-stained anatomical reference section with numbered structures (right). *Adamts2* expression is broadly detected in connective tissue-rich and mesenchymal compartments, including the developing dermis and subcutaneous tissues, craniofacial mesenchyme, tissues surrounding the vertebral column and cartilaginous skeletal elements, and stromal regions of developing thoracic and abdominal organs.

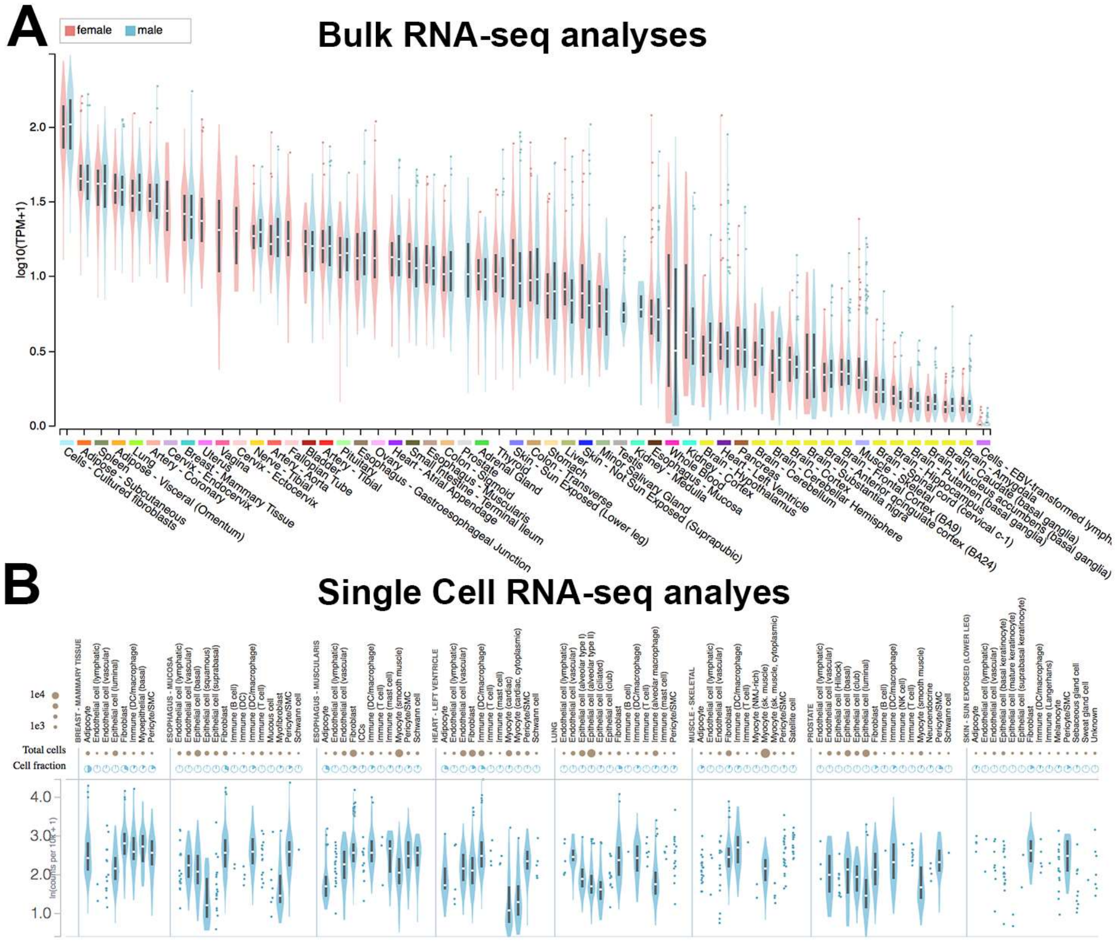

Figure S2. Tissue- and cell-type distribution of human ADAMTS2 expression. (A) Bulk RNA-sequencing analysis of ADAMTS2 expression across human tissues, separated by sex. Expression is shown as log10(TPM + 1), with violin plots representing the distribution of expression values and embedded box plots indicating median and interquartile range. ADAMTS2 expression is highest in cultured fibroblasts and is also prominent in multiple connective tissue-rich organs, including adipose tissue, arteries, lung, breast, uterus, vagina, gastrointestinal tissues, and musculoskeletal tissues. Female and male samples show broadly similar expression patterns. **(B)** Single-cell and single-nucleus RNA-sequencing analysis of ADAMTS2 expression across representative human tissues. Violin plots show cell-type-specific expression, while the annotations above each panel indicate the number of cells and proportion of cells expressing ADAMTS2. Expression is enriched in fibroblasts, myofibroblasts, smooth muscle cells, pericytes, and related mesenchymal populations, with comparatively low or inconsistent expression in most immune and neural cell types.

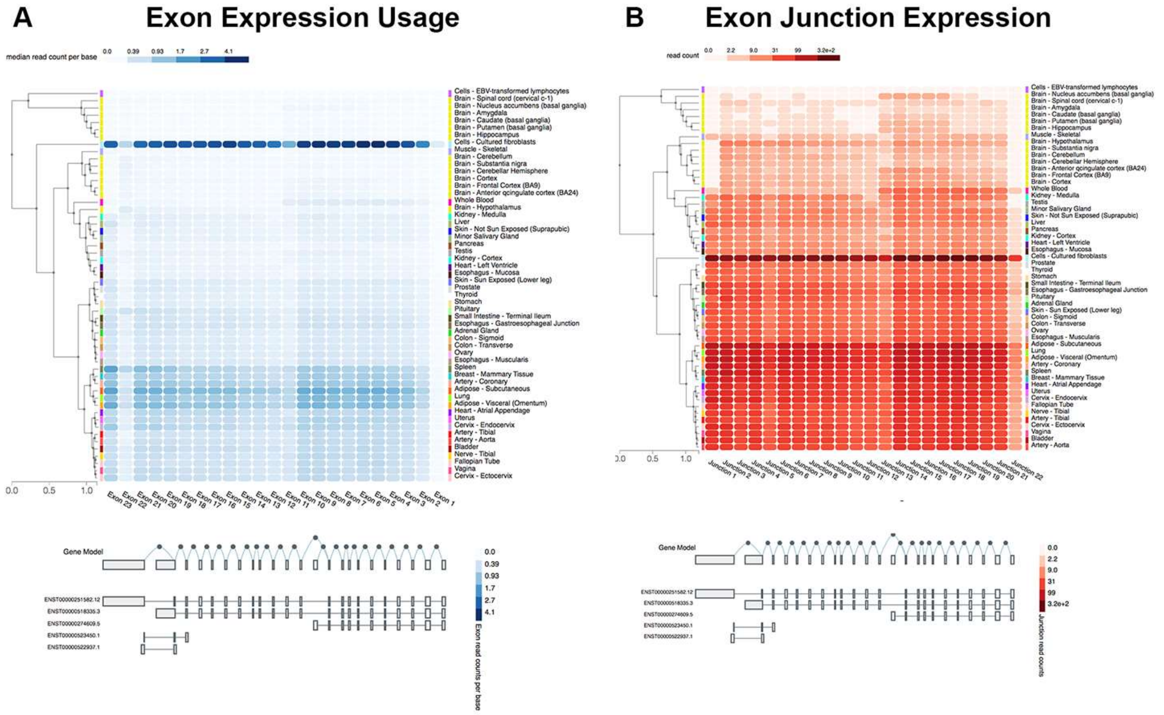

Figure S3. Exon-level and exon–exon junction expression of human ADAMTS2 across tissues. **(A)** Heatmap showing median read coverage per base across individual ADAMTS2 exons in human tissues. Rows represent tissues and columns represent exons, with the corresponding gene model and annotated transcript isoforms shown below. Cultured fibroblasts demonstrate the strongest and most continuous expression across the canonical coding exons, while lower but concordant exon usage is observed in multiple connective tissue-rich organs. **(B)** Heatmap showing read counts spanning sequential ADAMTS2 exon–exon junctions across human tissues. Junction usage generally parallels total transcript abundance and supports expression of a predominantly full-length ADAMTS2 transcript rather than extensive tissue-specific exon skipping. Dendrograms indicate hierarchical clustering of tissues based on exon or junction expression profiles.

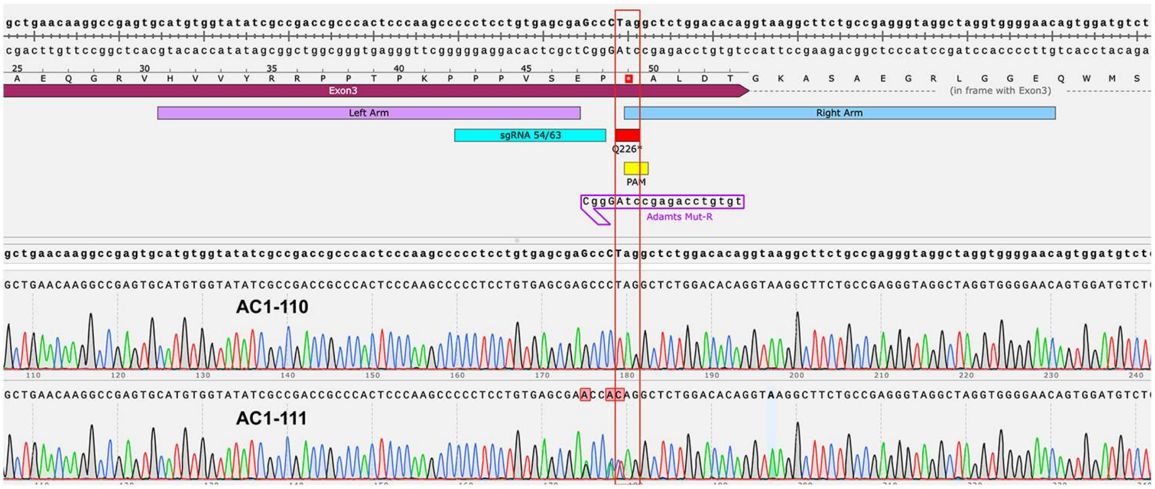

Figure S4. CRISPR/Cas9 targeting strategy and sequence validation of the Adamts2^{Q226} knock-in allele. Schematic illustrating the CRISPR/Cas9 approach used to introduce the patient-analog Q226* nonsense variant into the murine *Adamts2* gene. The guide RNA-targeting sequence and adjacent protospacer-adjacent motif are indicated above the genomic sequence, with the targeted nucleotide and engineered stop codon highlighted. The corresponding coding sequence and predicted amino acid translation are shown beneath the genomic locus. Representative Sanger sequencing chromatograms from two independently generated animals, AC1-110 (homozygous) and AC1-111 (heterozygous), confirm incorporation of the intended nucleotide substitution at the targeted site. Vertical lines indicate the edited nucleotide position, and the resulting codon change introduces a premature termination codon at amino acid 226.

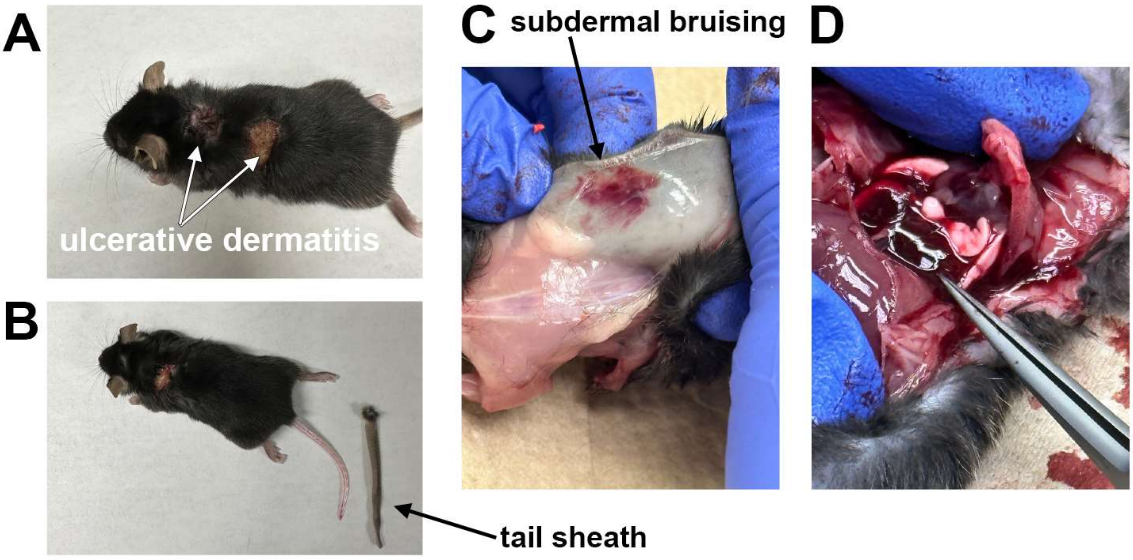

Figure S5. Gross connective tissue fragility phenotype of *Adamts2*$^{Q226*/Q226*}$ mice. (A) Representative homozygous mutant mouse showing severe ulcerative dermatitis, spontaneous skin wounds, and pronounced skin fragility over the dorsal shoulder region. **(B)** Separation of the tail skin from the underlying tissues during routine handling or euthanasia, producing a detached tail sheath and demonstrating marked loss of dermal integrity. **(C)** Reflection of externally intact-appearing skin reveals extensive subdermal bruising and hemorrhage, consistent with fragility of the dermal extracellular matrix and small blood vessels. **(D)** Abdominal dissection showing internal hemorrhage and blood accumulation surrounding abdominal organs, consistent with vascular or soft-tissue rupture.

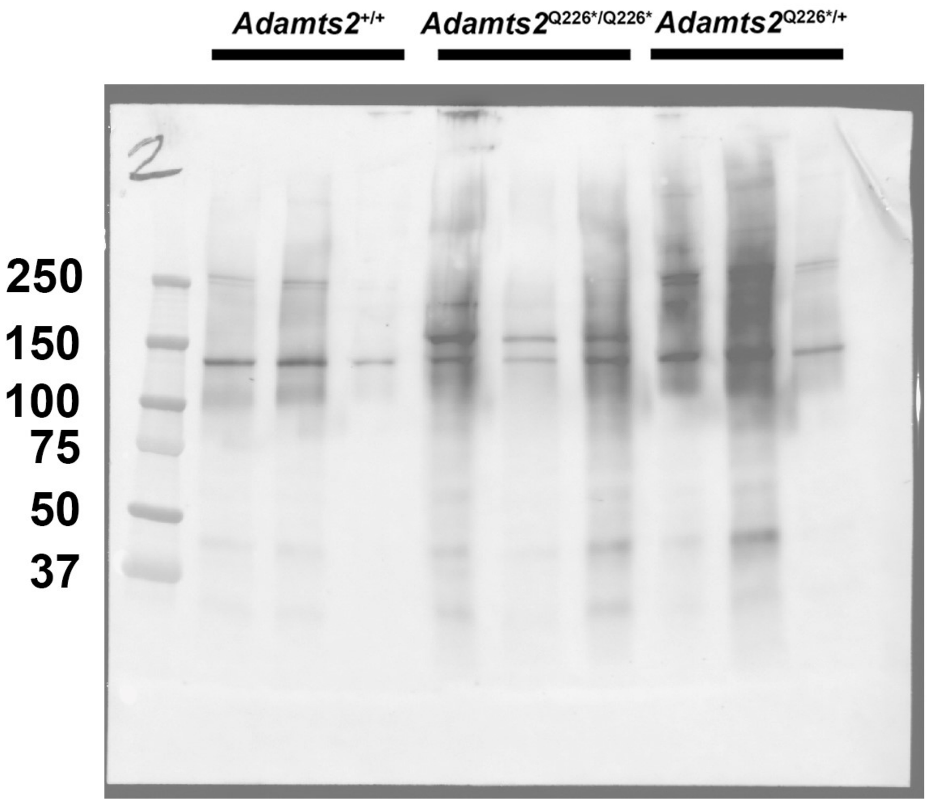

Figure S6: Raw, uncropped Western blot for collagen type I. Uncropped image from skin tissue lysate from the Adamts2 allelic series performed in triplicate. Molecular weights are designated on the left with the molecular weight ladder in the furthest to the left lane. Changes in unprocessed collagen (~150kDa) is apparent in the homozygous Adamts2Q226*/Q226* skin lysate. This image represents the corresponding full-length, uncropped blot for Figure 3 in the manuscript.

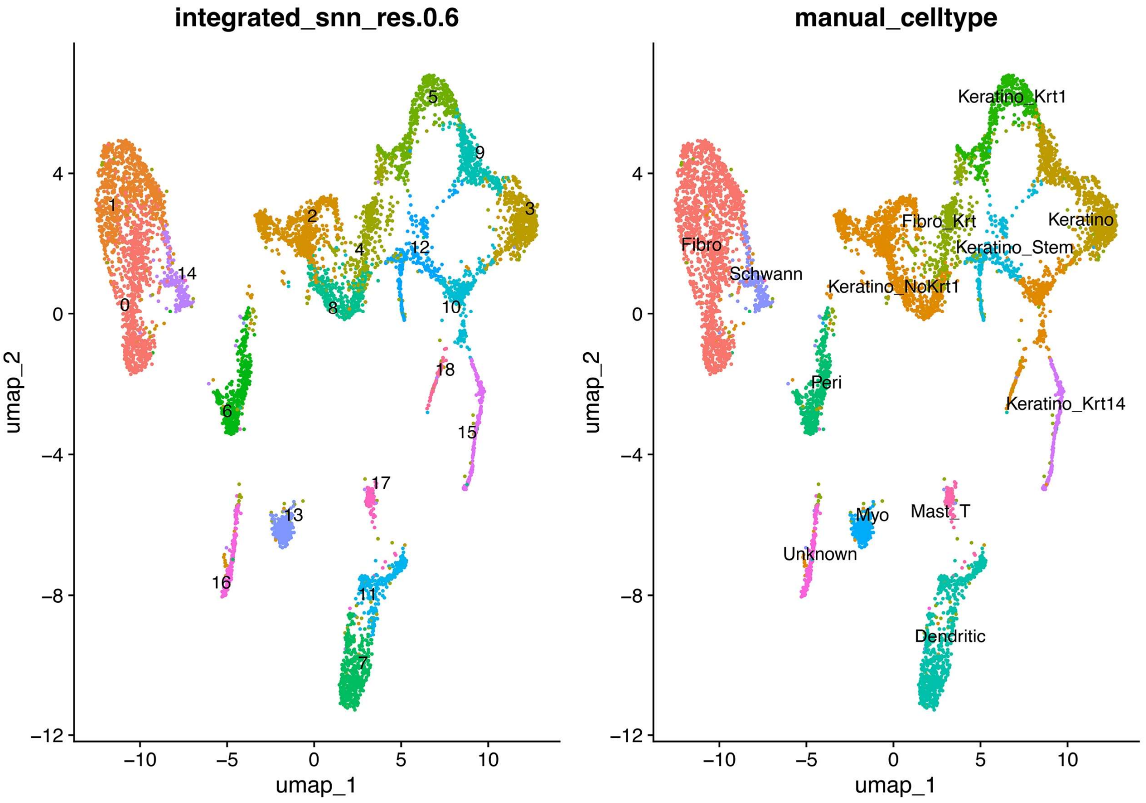

Figure S7. Single-nucleus transcriptomic identification and annotation of major skin cell populations. UMAP plots of integrated single-nucleus RNA-sequencing data from mouse skin. The left panel shows unsupervised clustering at a resolution of 0.6, with individual nuclei colored and labeled according to computationally defined clusters. The right panel shows the same integrated dataset annotated by manually assigned cell identities based on established lineage-specific marker expression. Identified populations included fibroblasts (Fibro), fibroblast-associated keratinocyte-like cells (Fibro_Krt), multiple keratinocyte subsets (Keratino, Keratino_Krt1, Keratino_NoKrt1, Keratino_Krt14, and Keratino_Stem), Schwann cells, pericytes (Peri), myocytes (Myo), mast cells/T cells (Mast_T), dendritic cells, and an unclassified population (Unknown). The separation of these transcriptionally distinct populations provided the cellular framework for subsequent differential expression, pathway-enrichment, and CellChat intercellular communication analyses.

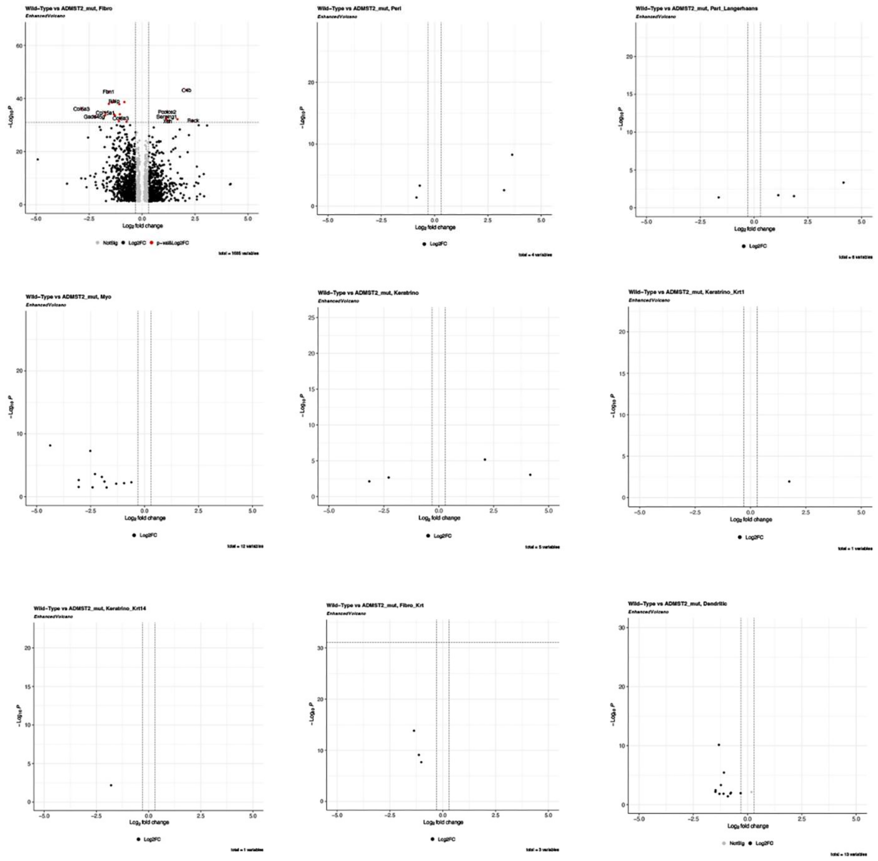

Figure S8. Cell type-resolved differential gene expression following ADAMTS2 deficiency. Volcano plots showing differential gene expression between wild-type and *Adamts2*$^{Q226*/Q226*}$ skin within individual single-nucleus RNA-sequencing cell populations, including fibroblasts, pericytes, Langerhans cells, myocytes, keratinocyte subsets, fibroblast-associated keratinocyte-like cells, and dendritic cells. The x-axis indicates $\log_2$ fold change and the y-axis indicates $-\log_{10}$ *P* value. Dashed vertical and horizontal lines denote the fold-change and statistical-significance thresholds used to identify differentially expressed genes. Fibroblasts exhibited the largest number and magnitude of transcriptional changes, including prominent alterations in extracellular matrix-associated and structural genes, whereas most other cell populations displayed comparatively few significant changes. These findings indicate that the transcriptional consequences of ADAMTS2 loss are concentrated primarily within the fibroblast compartment, with more limited cell type-specific effects across epithelial, vascular, neural, and immune populations.
