# Supplementary material for: An Adamts2 Knock-In Model of Dermatosparaxis Ehlers–Danlos Syndrome Reveals Defective Collagen Maturation
Source: Biomedicines. 2026 Aug 27;14(9):1928. doi: 10.3390/biomedicines14091928 (PMC13604185; doi:10.3390/biomedicines14091928)
Supplement: Supplementary file 1 [file biomedicines-14-01928-s001.zip › Supplemental Figures and Tables 082126/Table captions 081326.pdf]

Table S1. Quantitative digital pathology analysis of dermal collagen content, morphometry, and architecture across the *Adamts2* allelic series. Masson's trichrome-stained dorsal skin from wild-type (*Adamts2*$^{+/+}$), heterozygous (*Adamts2*$^{Q226*/+}$), and homozygous (*Adamts2*$^{Q226*/Q226*}$) mice was analyzed using the FibroNest digital pathology platform (n = 3 per genotype). Individual quantitative fibrosis traits are reported for collagen bulk, total collagen morphometry, fine-collagen morphometry, assembled-collagen morphometry, and fibrosis-associated tissue architecture. Measured features include collagen area, fiber number, branching, length, width, perimeter, density, tortuosity, eccentricity, solidity, orientation, and higher-order architectural descriptors. Composite scores summarize collagen content, overall morphometry, fine- and assembled-fiber morphometry, tissue architecture, and the integrated phenotypic fibrosis profile. Homozygous *Adamts2*$^{Q226*/Q226*}$ skin showed consistently reduced collagen composite, morphometric, architectural, and phenotypic fibrosis scores compared with wild-type and heterozygous skin, consistent with reduced collagen bulk, impaired fiber maturation, and loss of organized dermal matrix architecture. Heterozygous samples generally displayed intermediate or near-wild-type values.

Table S2. Cell type–specific differential gene expression in *Adamts2*$^{Q226*/Q226*}$ skin. Complete single-nucleus RNA-sequencing differential-expression results comparing dorsal skin from wild-type (*Adamts2*$^{+/+}$) and homozygous *Adamts2*$^{Q226*/Q226*}$ mice (n = 4 per genotype), stratified by annotated cell population. For each gene, the table reports the unadjusted P value (p_val), average log2 fold change (avg_log2FC), proportion of cells expressing the gene in each comparison group (pct.1 and pct.2), multiple-testing–adjusted P value (p_val_adj), difference in the proportion of expressing cells (diff), and corresponding cell type. Positive and negative avg_log2FC values indicate the direction of differential expression between genotypes.

Table S3. Significant cell type–specific differentially expressed genes in *Adamts2*$^{Q226*/Q226*}$ skin. Genes exhibiting significant differential expression between wild-type (*Adamts2*$^{+/+}$) and homozygous *Adamts2*$^{Q226*/Q226*}$ dorsal skin by single-nucleus RNA sequencing, stratified by cell type. Genes were considered significant at an adjusted P value < 0.05. The table reports the unadjusted P value (p_val), average log2 fold change (avg_log2FC), proportion of cells expressing each gene in the two comparison groups (pct.1 and pct.2), adjusted P value (p_val_adj), difference in the proportion of expressing cells (diff), and annotated cell type. The greatest number of significant transcriptional changes occurred within the fibroblast population, consistent with a fibroblast-centered response to loss of ADAMTS2 function.
